# *Brassicaceae* display variation in efficiency of photorespiratory carbon-recapturing mechanisms

**DOI:** 10.1093/jxb/erad250

**Published:** 2023-07-01

**Authors:** Urte Schlüter, Jacques W Bouvier, Ricardo Guerreiro, Milena Malisic, Carina Kontny, Philipp Westhoff, Benjamin Stich, Andreas P M Weber

**Affiliations:** Institute of Plant Biochemistry, Cluster of Excellence for Plant Sciences (CEPLAS), Heinrich Heine University, Universitätsstr. 1, D-40225 Düsseldorf, Germany; Institute of Plant Biochemistry, Cluster of Excellence for Plant Sciences (CEPLAS), Heinrich Heine University, Universitätsstr. 1, D-40225 Düsseldorf, Germany; Institute for Quantitative Genetics and Genomics of Plants, Cluster of Excellence for Plant Sciences (CEPLAS), Heinrich Heine University, Universitätsstr. 1, D-40225 Düsseldorf, Germany; Institute of Plant Biochemistry, Cluster of Excellence for Plant Sciences (CEPLAS), Heinrich Heine University, Universitätsstr. 1, D-40225 Düsseldorf, Germany; Institute of Plant Biochemistry, Cluster of Excellence for Plant Sciences (CEPLAS), Heinrich Heine University, Universitätsstr. 1, D-40225 Düsseldorf, Germany; Metabolomics and Metabolism Laboratory, Cluster of Excellence for Plant Sciences (CEPLAS), Heinrich Heine University, Universitätsstr. 1, D-40225 Düsseldorf, Germany; Institute for Quantitative Genetics and Genomics of Plants, Cluster of Excellence for Plant Sciences (CEPLAS), Heinrich Heine University, Universitätsstr. 1, D-40225 Düsseldorf, Germany; Institute of Plant Biochemistry, Cluster of Excellence for Plant Sciences (CEPLAS), Heinrich Heine University, Universitätsstr. 1, D-40225 Düsseldorf, Germany; MPI of Molecular Plant Physiology, Germany

**Keywords:** *Brassicaceae*, carbon-concentrating mechanisms, C_3_–C_4_ intermediates, C_2_ photosynthesis, photorespiration, photorespiratory glycine shuttle

## Abstract

Carbon-concentrating mechanisms enhance the carboxylase efficiency of Rubisco by providing supra-atmospheric concentrations of CO_2_ in its surroundings. Beside the C_4_ photosynthesis pathway, carbon concentration can also be achieved by the photorespiratory glycine shuttle which requires fewer and less complex modifications. Plants displaying CO_2_ compensation points between 10 ppm and 40 ppm are often considered to utilize such a photorespiratory shuttle and are termed ‘C_3_–C_4_ intermediates’. In the present study, we perform a physiological, biochemical, and anatomical survey of a large number of *Brassicaceae* species to better understand the C_3_–C_4_ intermediate phenotype, including its basic components and its plasticity. Our phylogenetic analysis suggested that C_3_–C_4_ metabolism evolved up to five times independently in the *Brassicaceae*. The efficiency of the pathway showed considerable variation. Centripetal accumulation of organelles in the bundle sheath was consistently observed in all C_3_–C_4_-classified taxa, indicating a crucial role for anatomical features in CO_2_-concentrating pathways. Leaf metabolite patterns were strongly influenced by the individual species, but accumulation of photorespiratory shuttle metabolites glycine and serine was generally observed. Analysis of phospho*enol*pyruvate carboxylase activities suggested that C_4_-like shuttles have not evolved in the investigated *Brassicaceae*. Convergent evolution of the photorespiratory shuttle indicates that it represents a distinct photosynthesis type that is beneficial in some environments.

## Teaser

Our physiological, biochemical, and anatomical survey of *Brassicaceae*suggests multiple evolution of C_3_–C_4_phenotypes connected to variation in photorespiratory carbon-recapturing efficiency and a distinct C_3_–C_4_bundle sheath anatomy.

## Introduction

The majority of plant species on Earth, including many crops, employ C_3_ photosynthesis. In these plants, under the present environmental conditions, the central photosynthetic enzyme Rubisco fixes approximately one molecule of oxy­gen for every three molecules of CO_2_ (Sharkey, 1988). Here, whilst the carboxylase reaction of Rubisco produces two molecules of 3-phosphglycerate (3PGA) which feeds into the Calvin–Benson–Bassham cycle (CBB), the oxygenase reaction produces 2-phosphoglycolate (2PG). 2PG is a competitive inhibitor of some CBB enzymes (Flügel *et al.*, 2017) and hence must be rapidly removed. Further, carbon contained in 2PG must be recycled into 3PGA to prevent depletion of CBB intermediates. These functions are fulfilled by the photorespiratory pathway. Photorespiration consists of coordinated enzyme activities that are located in different cellular compartments. In the plastids, 2PG is converted into glycolate followed by oxidation and transamination in the peroxisome, producing glycine. Glycine is transported into the mitochondria and metabolized into serine by glycine decarboxylation. Serine is finally converted into glycerate in the peroxisome and into 3PGA in the plastid. During glycine decarboxylation, previously fixed carbon and nitrogen are converted into CO_2_ and NH_3_, respectively. The re-fixation of carbon and nitrogen into organic forms, however, requires energy. Therefore, photorespiration is often considered a wasteful process in terms of energy, carbon, and nitrogen balance. Further, the affinity of Rubisco for O_2_ increases with rising temperatures; in addition, stomatal closure during water shortages can lead to a drop in CO_2_ concentrations inside the leaf. Thus, climate change could contribute to increased activity of the Rubisco oxygenase reaction.

Given the negative impact of photorespiration on plant productivity, there has been considerable interest in reducing flux through this pathway. For instance, various avenues to reduce photorespiratory losses which are being explored include increasing the capacity of the plant to recapture photorespiratory CO_2_, modifying Rubisco kinetic properties, and introducing carbon-concentrating mechanisms to limit the oxygenase reaction of Rubisco by creating an CO_2_-rich environment around the enzyme (Busch *et al.*, 2013). A better understanding of naturally occurring carbon-concentrating mechanisms will help in the design of these biotechnological approaches.

In C_3_ species, photosynthesis and photorespiration mainly take place in the mesophyll (M) of the leaves. Recapture of photorespiratory CO_2_ can be facilitated by arrangement of a continuous layer of plastids at the cell periphery next to the intercellular space. This arrangement acts to block diffusion of CO_2_ out of the cell because the CO_2_ would need to pass through the plastids where it can be reassimilated by Rubisco (Sage and Sage, 2009; Busch *et al.*, 2013). In rice, Rubisco-containing extensions (stromules) can increase the area of the plastidial barriers, preventing the efflux of CO_2_ produced in the mitochondria (Sage and Sage, 2009). C_3_ species such as wheat and rice achieve photorespiratory reassimilation rates of 24–38% (Busch *et al.*, 2013).

The number of chloroplasts in the C_3_ bundle sheath (BS) varies between species (Leegood, 2008), but they tend to be smaller and fewer in number compared with the M. The contribution of BS chloroplasts to leaf photosynthesis is considered to be small (Kinsman and Pyke, 1998; Janacek *et al.*, 2009; Aubry *et al.*, 2014). Nevertheless, BS cells possess important roles in leaf hydraulics, phloem loading, intra-leaf signalling, and transport processes (Leegood, 2008; Aubry *et al.*, 2014; Lundgren *et al*., 2014). Increases in BS organelle numbers indicate enhanced photosynthetic and photorespiratory activity. The centripetal arrangement of BS organelles helps to reduce loss of photorespiratory CO_2_ (Muhaidat *et al.*, 2011; Sage *et al.*, 2014). Such BS cells have also been labelled as ‘activated’ or ‘proto-Kranz’ anatomy (Gowik and Westhoff, 2011; Sage *et al*., 2014).

Further increases in BS CO_2_ concentration are possible by shifting the glycine decarboxylation step from the M to the BS (Monson *et al.*, 1984; Rawsthorne *et al.*, 1988a; Sage *et al*., 2014). This rearrangement forces photorespiratory glycine produced in the M to diffuse to the BS, where the tissue-specific increase in glycine decarboxylation activity promotes an elevated concentration of CO_2_ around the BS Rubisco and thus supresses its oxygenase reaction. BS-localized activity of glycine decarboxylation is mainly associated with cell specificity of the glycine decarboxylase (GDC) P-protein (GLDP) (Rawsthorne *et al.*, 1988a; Schulze *et al.*, 2013). The installation of a glycine shuttle is accompanied by a further increase in organelle numbers in the BS. The majority of mitochondria are therefore located between the centripetally arranged plastids and the vein-orientated cell wall (Sage *et al*., 2014). Such a combination of carbon supply by the glycine shuttle and efficient CO_2_ scavenging by an adequate organelle arrangement improves the leaf carbon conservation and can be measured as reduction in the carbon compensation point (CCP or Г). Plants employing the photorespiratory glycine shuttle are often classified as C_2_ species or C_3_–C_4_ intermediates of type I (Edwards and Ku, 1987; Sage *et al*., 2014). Their CO_2_ reassimilation capacity was estimated to be ~73% in *Moricandia arvensis* (Hunt *et al.*, 1987).

GDC activity is thought to be absent or considerably reduced in the M cells of well-developed C_2_ species, perhaps as a result of a loss-of-function mutation or insertion of a transposable element early in C_2_ evolution (Rawsthorne, 1992; Sage *et al*., 2012; Adwy *et al.*, 2015). Consistently, preferential BS localization of GLDP has been observed in many well-developed C_2_ species (Lundgren, 2020; Schlüter and Weber, 2020). The assimilatory power of the BS can generally be further enhanced by decarboxylation of additional metabolites. Organelle accumulation and enhancement of organellar metabolism in the BS could increase the availability of such compounds. The glycine shuttle not only transports carbon between the M and BS, but also creates a nitrogen imbalance between these cells where adjustment of leaf nitrogen metabolism in C_3_–C_4_ intermediates was proposed to occur by additional metabolite shuttling between M and BS cells (Mallmann *et al.*, 2014). Shuttling of malate, aspartate, pyruvate, α-alanine, α-ketoglutarate, and glutamate could contribute to rebalancing of nitrogen and energy balances between the two cell types (Mallmann *et al.*, 2014; Johnson *et al.*, 2021).

In the M cells, a rise in phospho*enol*pyruvate carboxylase (PEPC) activity can contribute to the provision of these shuttle metabolites. PEPC fixes carbon by catalysing the addition of bicarbonate to phospho*enol*pyruvate (PEP), forming the C_4_ acid oxaloacetate that is usually quickly converted into malate or aspartate. In contrast to Rubisco, PEPC possesses higher substrate specificity and affinity. Combined with decarboxylation reactions in the BS, high PEPC activity in the M cells can implement a carbon shuttle mechanism transporting C_4_ metabolites into the BS where CO_2_ is released. Plants using the glycine shuttle in combination with such a C_4_ shuttle have been identified mainly in the *Asteraceae* genus *Flaveria*. They are also termed C_2_+C_4_ or C_3_–C_4_ type II species (Edwards and Ku, 1987; Sage *et al*., 2014; Bellasio, 2017).

Additional anatomic rearrangements and consequent separation of the PEPC and Rubisco reactions into the M and BS cells finally support an efficient C_4_ cycle (Taniguchi *et al.*, 2021). In the M cells, CO_2_ is converted into bicarbonate by carbonic anhydrase and is then fixed by PEPC. The bound carbon diffuses into the BS mainly in the form of malate or aspartate. Decarboxylation is then mediated by the NADP-malic enzyme in plastids, NAD-malic enzyme in the mitochondria, or phospho*enol*pyruvate carboxykinase in the cytosol. The cycle is completed by diffusion of a C_3_ metabolite back to the M cells where ATP is needed for PEP regeneration by pyruvate phosphate dikinase. Plants with a strong C_4_ shuttle, but which still exhibit Rubisco in the M, are classified as C_4_-like (Moore *et al.*, 1989). In bona fide C_4_ species, all CO_2_ is first assimilated by PEPC, with subsequent shuttling of the resulting C_4_ acid to the BS where CO_2_ is then delivered to Rubisco by a decarboxylase reaction. High CO_2_ partial pressure in the BS strongly represses the oxygenase reaction, the following photorespiratory pathway, and the concomitant loss of CO_2_ and NH_3_.

The efficiency of the C_4_ shuttle also depends on anatomical features, especially the close connection between M and BS cells. In the majority of C_4_ species, the BS forms a tight cell layer around the veins without direct exposure to the intercellular space (Sage *et al*., 2014). The proportion of M tissue is reduced to a second cell layer around the BS cells, and C_4_ species usually have high vein densities. Since CO_2_ fixation in C_4_ species can continue at lower internal CO_2_ concentration (*C*i) and stomatal conductance, water use efficiency (WUE) is improved compared with C_3_ species. Operation of Rubisco under high CO_2_ partial pressure allows high efficiency for the carboxylase reaction with lower amounts of protein, thus also improving the nitrogen use efficiency of C_4_ photosynthesis.

The continuous fitness gain in the intermediate forms seems to have been an important prerequisite for the evolution of the complex C_4_ biochemistry and anatomy (Heckmann *et al.*, 2013; Williams *et al.*, 2013; Mallmann *et al.*, 2014; Blätke and Bräutigam, 2019; Dunning *et al.*, 2019a). Species using the photorespiratory glycine shuttle have been identified in various monocot and dicot plant lineages, often but not always in phylogenetic proximity to C_4_ species (Sage *et al*., 2011). Convergent evolution of the C_3_–C_4_ pathway indicates substantial improvement of leaf carbon economy at least under certain environmental conditions (Bellasio and Farquhar, 2019; Lundgren, 2020). Reduction in the CCP and centripetal accumulation of BS organelles seem to be general features of C_3_–C_4_ plants, but knowledge about the anatomical and biochemical plasticity of the pathway and their influence on leaf physiological is still limited (Schlüter and Weber, 2016).

In our study, we concentrated on the *Brassiceae* tribe that evolved ~23 million years ago in the circum-Mediterranean region (Arias and Pires, 2012). It includes multiple lineages of C_3_–C_4_ intermediates, but no known C_4_ species (Apel *et al.*, 1997). Aiming at large sample sizes from the group of C_3_ and well as C_3–_C_4_ species, we selected taxa from all currently known C_3_–C_4_ intermediates and related C_3_ species from the genera *Moricandia*, *Diplotaxis*, and *Brassica*. Among the *Brassiceae* are also numerous crops species such as canola or rapeseed (*Brassica napus*), cabbage (*Brassica oleracea*), radish (*Raphanus sativus*), mustard (*Sinapis alba*), and the salad vegetable rocket or arugula (*Eruca sativa*, *Diplotaxis tenuifolia*). With the exception of *D. tenuifolia*, these are all C_3_ crops.

In the present study, we analysed 34 taxa representing 28 *Brassicaceae* species. Our investigation of taxa from diverse photosynthesis types allowed us to assess the variation in 75 photosynthetic-related parameters across C_3_ and C_3_–C_4_ species. The *Cleomaceae Gynandropsis gynadra* was included for comparison with the anatomy, biochemistry, and physiology of C_4_ species. We use the CCP to rank the species and accessions according to their carbon-concentrating capacity. By parallel analysis of leaf structural features, we investigate the correlation between carbon-concentrating capacity and vein density, leaf thickness, and organelle arrangement in the BS. As the installation of the glycine shuttle requires further metabolic adjustments in the leaf such as the nitrogen balancing between M and BS cells, the primary metabolite pattern of the leaf sections was also analysed. Consequences of the anatomical and biochemical changes for the leaf physiology including assimilation and WUE were assessed. The large number of plant taxa will allow us also to learn about lineage-specific developments of the C_3_–C_4_ pathways and potential variation within the trait. Detailed knowledge about the carbon-concentrating mechanisms existing in *Brassiceae* can help to identify interesting traits for engineering or breeding approaches.

## Materials and methods

### Plant cultivation

All seeds used in this study were obtained from either Botanical gardens, seed stock centres, or seed companies. Since physiology can vary between populations from the same species (Lundgren *et al*., 2016; Yorimitsu *et al*., 2019; Gomez *et al.*, 2020), multiple accessions were used from some species. The complete list of plant taxa comprised: *Arabidopsis thaliana* (L.) Heynh. (At), *Brassica gravinae* Ten. four accessions, Bg1, Bg2, Bg3, and Bg4), *Brassica juncea* (L.) Czern. (Bj), *Brassica napus* L. (Bn), *Brassica nigra* (L.) W.D.J. Koch subsp. *nigra* var nigra (Bni), *Brassica oleraceae* L. (Bo), *Brassica rapa* L. (Br), *Brassica repanda* (Willd.) (Be), *Brassica tournefortii* Gouan. (two accessions, Bt1 and Bt2), *Carrichtera annua* (L.) DC. (Ca), *Diplotaxis acris* Boiss. (Da), *Diplotaxis erucoides* (L.) DC. (De), *Diplotaxis harra* Boiss. (Dh), *Diplotaxis muralis* (L.) DC. (Dm), *Diplotaxis tenuifolia* (L.) DC. (Dt), *Diplotaxis tenuisiliqua* Delile (Ds), *Diplotaxis viminea* (L.) DC. (Dv), *Eruca sativa* Mill. (Es), *Hirschfeldia incana* (L.) Lagr.-Foss (two accessions HIR1 and HIR3), *Moricandia arvensis* (L.) DC. (Ma), *Moricandia moricandioide*s (Boiss.) Heywood (Mm), *Moricandia nitens* E.Durand & Barratte (Mn), *Moricandia sinaica* Boiss. (Msi), *Moricandia spinosa* Pomel (Mp), *Moricandia suffruticosa* (Desf.) Coss. & Durieu (Ms), *Raphanus raphanistrum* L. (Rr), *Raphanus sativus* subsp. *sativus* (L.) (Rs), and *Sinapis alba* L. (Sa). From the *Cleomaceae*, the C_4_ species *Gynandropsis gynandra* (L.) briq. (Gg) was also included in the present study. A complete list of origins for these seeds can be found in Supplementary Table S1.

All seeds were vapour sterilized by incubation in an exicator with a fresh mixture of 100 ml of 13% Na-hypochloride with 3 ml of 37% HCl for 2 h. The sterilized seeds were then germinated on plates containing 0.22% (w/v) Murashige and Skoog medium, 50 mM MES pH 5.7, and 0.8% (w/v) agar. After 7–10 d, the seedlings were transferred to pots containing a mixture of sand and soil (Floraton 1 soil mixture, Floraguard, Germany) at a ratio of 1:2. All plants were firstly cultivated in climate chambers (CLF Mobilux Growbanks, Germany) under 12 h day conditions with 23 °C/20 °C day/night temperatures and ~200 µmol s^–1^ m^–2^ light. After establishment in soil, 2-week-old plants were transferred to the greenhouse of the Heinrich Heine University with a 16 h day/8 h night cycle. Natural light conditions in the greenhouse were supplemented with metal halide lamps (400 W, DH Licht, Germany) so that the plants received between 250 µmol m^–2^ s^–1^ and 400 µmol m^–2^ s^–1^. Minimum temperatures of the greenhouse were controlled to 21 °C during the night and 24 °C during the day.

The initial main experiment was conducted between October 2018 and March 2019. A small number of additional accessions were also studied between July and October 2020 following the same protocol. As controls, *G. gynandra*, *D. tenuifolia*, and *H. incana* (HIR3) were included in both experiments. Gas exchange parameters obtained for these three species, especially CCPs, were stable across the experiments. Thus, results from both experiments were considered comparable. Gas exchange was measured on the youngest fully expanded rosette leaves before onset of flowering. After gas exchange measurements were performed, plants were taken back to the greenhouse for 2 d. Following that, leaf material was harvested for metabolite analysis (only experiment in 2018/2019), elemental analyser isotope ratio mass spectrometry (EA-IRMS) analysis, leaf vein determination, and embedding for light microscopy. A third experiment was conducted on plant accessions selected from the initial experiments in September to November 2021 in the new greenhouses of the Heinrich-Heine University equipped with natural light LED lamps using the same experimental design. Leaves were snap-frozen in liquid nitrogen directly in the greenhouse in the late morning Samples for protein and PEPC assay were snap-frozen in liquid nitrogen at midday. An additional leaf was used for determination of specific leaf area (SLA). For the majority of taxa, 4–6 plants were analysed as replicates in the different experiments; for a few taxa, only two plants were available for analysis. In the graphs, each data point represents a measurement from a single plant.

### Phylogenetic inference

Plants for genome sequencing were grown in a climate-controlled chamber from the same seed stock used for physiological analysis. Linked read sequencing was performed by 10× Genomics and BGI, complemented by PacBio and Nanopore long read sequencing for some species (Guerreiro *et al.*, 2023). Most assemblies are linked read assemblies, some being scaffolded and gap-filled with the long read data, while two assemblies are long read assemblies polished and scaffolded by linked reads.

The assemblies are pseudohaploid, with alternative haplotype contigs having been removed with Purge Haplotigs (v1.1.0) (Roach *et al.*, 2018). The novel genome assemblies were complemented with literature assemblies (Guerreiro *et al.*, 2023). Repeat regions were identified for each assembly with Mite-hunter (Han and Wessler, 2010), genometools (v1.5.9), LTRharvest (Ellinghaus *et al.*, 2008), and RepeatModeller (v1.0.11) (Smit *et al.*, 2015). Those repeat regions were masked out of the assembly prior to gene annotation using RepeatMasker (v4.0.9) (Smit *et al.*, 2015). Gene annotation was performed using Maker2 (Campbell *et al.*, 2014) and the same protein database for every genome (Guerreiro *et al.*, 2023). The predicted proteomes of every species were filtered for annotation edit distance (AED; Eilbeck *et al.*, 2009) values <0.5. Functional annotation was performed with AHRD in order to remove transposon-related genes (Guerreiro *et al.*, 2023).

Finally, the filtered proteomes were fed into Orthofinder v2.5.1 (Emms and Kelly, 2015, 2019) for orthology identification based on all versus all sequence BLASTp searches and MCL clustering (Emms and Kelly, 2015). Multiple sequence alignments for identified orthogroups (HOGs) were produced with MAFFT and used for creating gene trees with RaxML with the PROTGAMMALG substitution model. The gene trees of HOGs with single-copy genes for at least 80% of species (102 HOGs) were fed to ASTRAL-pro (Zhang *et al.*, 2020) for the creation of a species phylogeny with quartet-based local posterior probability values (Sayyari and Mirarab, 2016) for each node.

### Photosynthetic gas exchange

Gas exchange was measured on the youngest fully expanded rosette leaf ~6–10 weeks after sowing and before the onset of flowering. The settings of the LI-6800 (LI-COR, Lincoln, NE, USA) were as follows: flow of 300 µmol s^–1^, fan speed of 10 0000 rpm, light intensity of 1500 µmol m^–2^ s^–1^, leaf temperature of 25 °C, and vapour pressure deficit of 1.5 kPa. After adjustment of leaves to the conditions in the leaf chamber, *A*–*C*i curves were measured at reference atmospheric CO_2_ concentrations of 400, 200, 100, 75, 50, 40, 30, 20, 10, 0, 400, 400, 600, 800, 1200, and 1600 ppm. For the experiment in 2018/2019, the LI-6800 was equipped with a fluorescence head measuring *F*_v_ʹ/*F*_m_ʹ and electron transport rate (ETR) at each CO_2_ level.

For calculation of the CCP and the carboxylation efficiency (CE=initial slope of *A*–*C*i), a minimum of four data points in the linear range close to the interception with the *C*i axis were used. Maximal assimilation was determined at CO_2_ concentrations between 1200 ppm and 1600 ppm. Assimilation (*A*), stomatal conductance (*g*_sw_), *C*i, WUE=*A*/*g*_sw_, and the ratio between internal and external CO_2_ concentrations (*C*i/*C*a) from the measurements at 400 (ambient CO_2_), 200, 100, and 50 ppm CO_2_ were used for more detailed physiological analysis of the investigated plant accessions.

### Metabolite and element analysis

After the gas exchange measurements, plants were allowed to readjust to greenhouse conditions before sampling for metabolite patterns. Leaves were snap-frozen in liquid nitrogen directly in the greenhouse in the late morning and stored at –80 °C. The leaf samples then were homogenized into a fine powder by grinding in liquid nitrogen. Soluble metabolites were extracted in 1.5 ml of extraction solution consisting of water:methanol:chloroform in a 1:2.5:1 mixture following the method of Fiehn *et al.* (2000). A 30 µl aliquot of the supernatant was dried completely in a vacuum concentrator and derivatized for GC-MS measurements (Gu, 2012). GC-MS measurements were performed as described by Shim *et al.* (2020) using a 5977B GC-MSD (both Agilent Technologies). Metabolites were identified via MassHunter Qualitative (v b08.00, Agilent Technologies) by comparison of spectra with the NIST14 Mass Spectral Library (https://www.nist.gov/srd/nist-standard-reference-database-1a-v14). A standard mixture containing all target compounds at a concentration of 5 µM was processed in parallel to the samples as a response check and retention time reference. Peaks were integrated using MassHunter Quantitative (v b08.00, Agilent Technologies). For relative quantification, all metabolite peak areas were normalized to the corresponding fresh weight used for extraction and to the peak area of the internal standard ribitol or dimethylphenylalanine (Sigma-Aldrich). The same homogenized leaf material was used for determination of δ^13^C and CN ratios. After lyophilization, the material was analysed using an Isoprime 100 isotope ratio mass spectrometer coupled to an ISOTOPE cube elemental analyser (both from Elementar, Hanau, Germany) according to Gowik *et al.* (2011).

### Vein density measurements

The top third of mature rosette leaves were used for vein density measurements. The leaf material was cleared in an acetic acid:ethanol mix (1:3) overnight followed by staining of cell walls in 5% safranin O in ethanol, and de-staining in 70% ethanol. Pictures were taken using a Nikon eclipse Ti-U microscope equipped with a ProgRes MF camera from Jenoptik, Germany, at ×4 magnification. The vein density was analysed with ImageJ software determining the total vein length per total micrograph area. In most cases, six leaves were analysed for vein density per line with a minimum of three pictures measured and averaged per leaf.

### Specific leaf area

Whole mature rosette leaves were cut and their outlines were copied on checked paper. The FW was measured immediately after and DW was subsequently determined after 48 h at 60 °C. The leaf area was determined using ImageJ software. For calculation of SLA, the area was divided by the dry weight.

### Analysis of leaf cross-section

For light microscopy, sections of ~1 × 2 mm were cut from the top third of mature rosette leaves and immediately fixed in 2% paraformaldehyde, 2% glutaraldehyde, 0.1% Triton X-100 in phosphate-buffered saline (PBS; 137 mM NaCl, 2.7 mM KCl, 12 mM H_2_PO_4_^–^/HPO_4_^2–^, pH 7.4). A vacuum was applied to the reaction tubes until all leaf sections sank to the bottom. The sections were incubated in the primary fixation solution overnight followed by washing once with PBS solution, pH 7.4, and twice with distilled H_2_O. For post-fixation, the sections were incubated in 1% OsO_4_ for 45 min followed by washing again three times with distilled H_2_O. A dehydration series was performed ranging from 30% to 100% acetone, followed by incubation in increasing proportions of Araldite resin until 100% Araldite was reached. The sections were finally positioned into flat embedding moulds and polymerized at 65 °C for at 48 h.

After cutting, the leaf sections were stained with toluidine blue solution (0.5% toluidine blue, 0.5% methylene blue, 6% Na_2_B_4_O_7_, 1% H_3_BO_3_) and studied under a light microscope (Zeiss Axio Observer, Carl Zeiss, Germany). For quantitative analysis, pictures of at least three BSs per biological replicate were taken and analysed with ImageJ software. The following parameters were determined per BS for quantitative analysis: cross-section area of the BS cell (BS_cell_area), area of organelles orientated towards the vein (V_organelle_area), area of organelles orientated towards the intercellular space (ICS), and M (M_organelle_area). The following parameter were calculated: percentage of vein-orientated organelle area per BS cell area (percent_V_organelle), percentage of organelle area orientated towards the ICS and M (percent_M_organelles), the total organelle area per cell (V_organelle_area + M_organelle_area = total_organelle_area), and the ratio between percentage values for vein and ICS/M-orientated organelles. Furthermore, the leaf thickness was measured at the site of the selected BS. Three representative cells were analysed per BS, and three BSs were analysed per plant.

### PEPC activity

Total soluble proteins were extracted from the homogenized leaf material in 50 mM HEPES-KOH pH 7.5, 5 mM MgCl_2_, 2 mM DTT, 1 mM EDTA, 0.5% Triton X-100. For the PEPC assay, 10 µl of the extract were mixed with assay buffer consisting of 100 mM Tricine-KOH pH 8.0, 5 mM MgCl_2_, 2 mM DTT, 1 mM KHCO_3_, 0.2 mM NADH, 5 mM glucose-6-phosphate, and 2 U ml^–1^ malate dehydrogenase in a microtitre plate. The reaction was started after addition of PEP to a final concentration of 5 mM in the assay. Protein content of the solutions was determined with the BCA assay (Thermo Fischer Scientific).

### Statistical analysis

Data analysis was performed using R (www.R-project.org). Statistical differences between the measured parameters in the accessions were calculated by one-way ANOVA followed by Tukey’s post-hoc test. Differences between parameters in C_3_ and C_3_–C_4_ photosynthesis types were determined by a two-tailed *t*-test.

## Results

### Assessment of CO_2_-concentrating efficiency by measuring CO_2_ compensation points

**Fig. 1.**
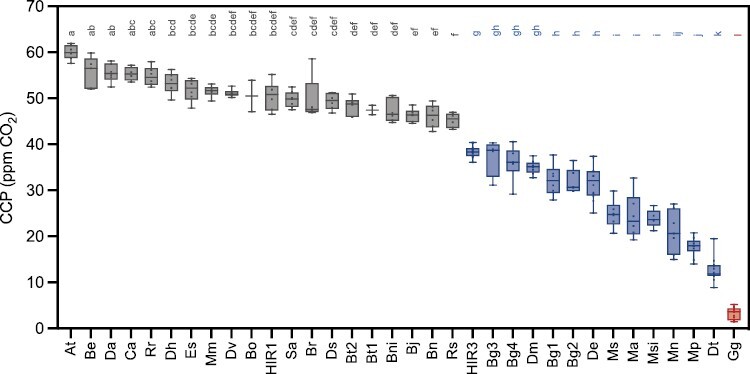
CO_2_ compensation points in selected *Brassicaceae*. CO_2_ compensation points were measured in young, fully expanded leaves of greenhouse-grown plants. The letters above each box indicate the statistical grouping determined by ANOVA followed by HSD post-hoc test with α=0.05. The tested taxa are coloured according to photosynthesis type as C_3_ (grey), C_3_–C_4_ (blue), and C_4_ (red). Plant names have been abbreviated for legibility and are provided in full in the Materials and methods.

The CCP is a measure of the internal leaf CO_2_ level at which photosynthetic CO_2_ fixation is equal to the CO_2_ release by photorespiration, day respiration, and other catalytic processes (i.e. the concentration at which net CO_2_ assimilation is zero). In the present study, the CCP was determined from *A*–*C*i curves across a diverse range of 33 *Brassicaceae* taxa representing 28 species to assess their carbon usage efficiency (Fig. 1).

When sorting all sampled taxa in the *Brassicaceae* according to their CCP, a range of CCP values from 60 ppm to 12 ppm was detected. The *Cleomaceae* C_4_ species *G. gynandra* with highly efficient CO_2_ concentration showed CCP values <10 ppm. Plants with CCP values between 10 ppm and 40 ppm are predicted to utilize less efficient CO_2_-concentrating mechanisms, such as the photorespiratory shuttle, and were hereby classified as C_3_–C_4_ intermediates. In contrast, all accessions and species with a CCP >40 ppm were classified as C_3_ species. This grouping was supported by ANOVA with post-hoc Tukey’s HSD test (α=0.05; Fig. 1). The same threshold values were also used in the survey by Krenzer *et al.* (1975).

In our study, *A. thaliana* exhibited the highest CCP of 60.1 ppm. Slightly lower CCPs between 45 ppm and 60 ppm were observed for the C_3_ species of the *Brassiceae* clade, including *B. repanda*, *D. acris*, *C. annua*, *R. raphanistrum*, *D. harra*, *E. sativa*, *M. moricandioides*, *D. viminea*, *B. oleraceae*, *H. incana* HIR1, *S. alba*, *B. rapa*, *D. tenuisiliqua*, *B. tournefortii*, *B. nigra*, *B. juncea*, *B. napus*, and *R. sativus.* In comparison with these C_3_ plants, a significant reduction in the CCPs was observed in 14 taxa classified in the present study as C_3_–C_4_ intermediates, these included *H. incana* HIR3, four taxa of *B. gravinae*, *D. muralis*, *D. erucoides*, *M. suffruticosa*, *M. arvensis*, *M. sinaica*, *M. nitens*, *M. spinosa*, and *D. tenuifolia*.

Among the C_3_–C_4_ intermediates, the lowest CCP value of 12 ppm was measured in *D. tenuifolia*. In contrast, the highest CCP value recorded among the C_3_–C_4_ intermediates at ~40 ppm was measured in *H. incana* HIR3. Importantly, the identification of this taxon as a C_3_–C_4_ intermediate species which presumably operates a CO_2_-concentrating mechanism is described here for the first time. Interestingly, another taxon assigned to the same species (*H. incana* HIR1) exhibited a CCP value within the range of C_3_ species (Fig. 1). In all other species, different taxa were assigned to the same photosynthetic type. Altogether, a wide range of CCPs was observed among *Brassicaceae* and especially the C_3_–C_4_ intermediates.

### Phylogeny suggests multiple origins of C _3_ –C _4_ photosynthesis in the *Brassiceae*

**Fig. 2.**
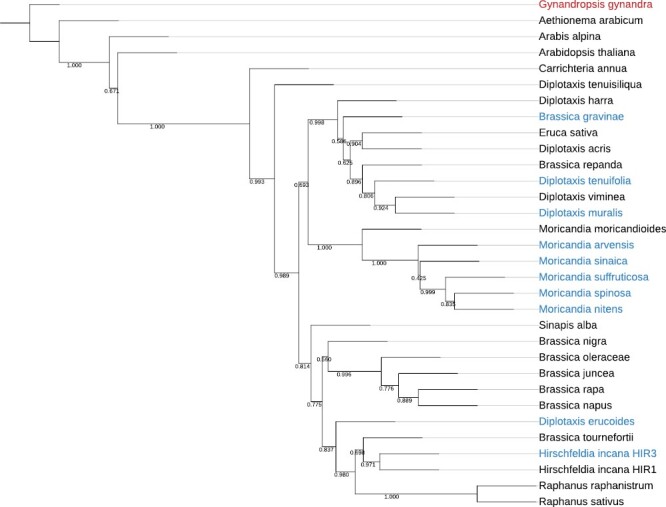
Phylogeny and photosynthesis types. The numbers on the nodes represents quartet-based local posterior probability values. Species names are coloured according to the photosynthesis type as C_3_ (grey), C_3_–C_4_ (blue), and C_4_ (red).

The phylogenetic relationship among the plant species selected for this study was investigated using sequence data from 102 orthogroups (Fig. 2). When investigating the distribution of species classified as C_3_–C_4_ intermediates based on CCP data, the tree reveals multiple origins of CO_2_-concentrating mechanisms in the *Brassiceae*. We are aware that the presented tree includes only a small subset of species from the *Brassiceae* group. However, a more densely sampled phylogenetic tree by Koch and Lemmel (2019) suggests the same number of origins because our predicted C_3_–C_4_ lineages all have common ancestors with C_3_ species.

The phylogenetic positions of *B. gravinae*, *H. incana* HIR3, and *D. erucoides* on the tree suggest independent origins of C_3_–C_4_ features. In the *Moricandia* group, C_3_–C_4_ features are observed among the close relatives *M. arvensis*, *M. suffruticosa*, *M. nitens*, *M. sinaica*, and *M. spinosa*, but not the sister species *M. moricandioides* which is C_3_. Thus, C_3_–C_4_ most probably evolved once in this group, in a common ancestor preceding the speciation of *M. arvensis*, *M. suffruticosa*, *M. nitens*, *M. sinaica*, and *M. spinosa* but after diversification from *M. moricandioides*. Finally, C_3_–C_4_-like CCPs were observed in *D. tenuifolia* and *D. muralis*. Both of these respective species are closely related to the C_3_ species *D. viminea*. *Diplotaxis muralis* is a natural hybrid between the C_3_ parent *D. viminea* and the C_3_–C_4_ parent *D. tenuifolia*. Therefore, the C_3_–C_4_ features in *D. muralis* are assumed to be inherited from *D. tenuifolia* (Ueno *et al.*, 2006). In summary, our phylogenetic data indicate that C_3_–C_4_ features have independently evolved up to five times in the *Brassicaceae*, in *B. gravinae*, in *D. erucoides*, in *H. incana* HIR3, in the *Moricandia* group, and in *D. tenuifolia*.

### Physiology of C _3_ , C _3_ –C _4_ , and C _4_ leaves under different CO _2_ concentrations

**Fig. 3.**
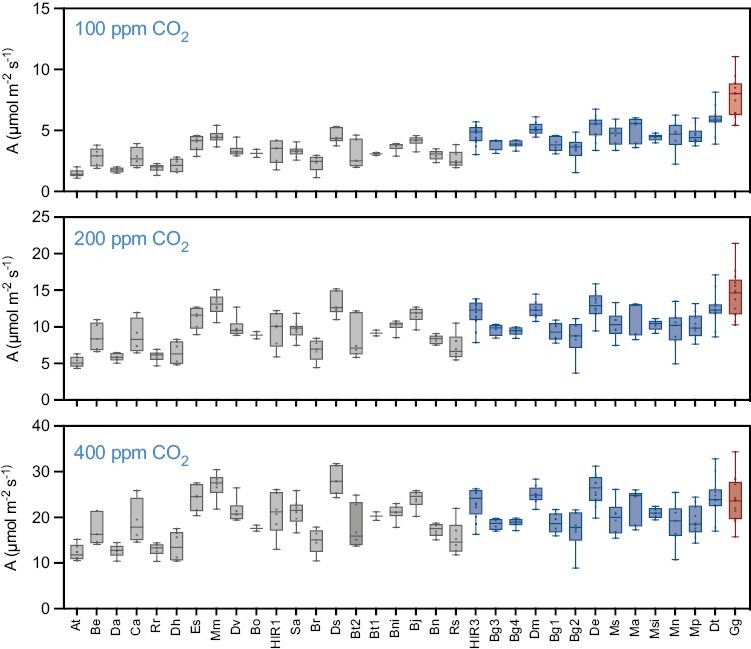
Net assimilations under different CO_2_ concentrations. Assimilation was measured under conditions of ambient CO_2_ (400 ppm) or reduced CO_2_ concentrations of 200 ppm and 100 ppm. The tested taxa were sorted according to their CO_2_ compensation points and coloured according to the photosynthesis type as C_3_ (grey), C_3_–C_4_ (blue), and C_4_ (red). Taxa names have been abbreviated for legibility and are provided in Fig. 2 and the Materials and methods.

Efficiency of photosynthetic gas exchange in the different C_3_ and C_3_–C_4_*Brassicaceae* was assessed under ambient CO_2_ (400 ppm) and saturating light (1500 µmol m^–2^ s^–1^). Here, under these conditions, no association between photosynthesis type and net assimilation could be observed (Fig. 3). For instance, in C_3_ plants, assimilation rates under ambient CO_2_ varied between 12.3 µmol m^–2^ s^–1^ (*A. thaliana*) and 28.1 µmol m^–2^ s^–1^ (*D. tenuisiliqua*) (Fig. 3; Supplementary Table S2), whilst among C_3_–C_4_ intermediates, assimilation rates varied between 17.3 µmol m^–2^ s^–1^ (*B. gravinae* accession 2) and 26.1 µmol m^–2^ s^–1^ (*D. erucoides*). Moreover, assimilation rates achieved in the C_4_ species *G. gynandra* of 23.7 µmol m^–2^ s^–1^ were similar to rates in non-C_4_ plants. Thus, assimilatory capacity under ambient CO_2_ conditions appears to be species specific, rather than determined by the activity of a metabolite shuttle mechanism.

In contrast to the above, enhanced rates of assimilation were discovered in plants operating a CO_2_-concentrating mechanism under lower atmospheric CO_2_ concentrations (Fig. 3; Supplementary Table S2). For instance, at pre-industrial levels of 200 ppm CO_2_, the C_4_*G. gynandra* showed higher assimilatory capacity compared with any other C_3_ or C_3_–C_4_ species (Fig. 3; Supplementary Table S2). Further, this elevated assimilation rate observed in *G. gynandra* became even more pronounced under 100 ppm CO_2_ (Fig. 3; Supplementary Table S2). Interestingly, C_3_–C_4_ intermediate species tended to perform better than C_3_ plants under subambient CO_2_ conditions (Fig. 3; Supplementary Table S2). On average, across all plants of each photosynthesis type, assimilation was significantly higher in the C_3_–C_4_ group compared with the C_3_ group under CO_2_ conditions of ≤200 ppm (*t*-test, *P*<0.05; Supplementary Fig. S1). Thus, although CO_2_-concentrating mechanisms do not improve net assimilation under present atmospheric CO_2_, they appear to be advantageous under former pre-industrial levels of CO_2_.

**Fig. 4.**
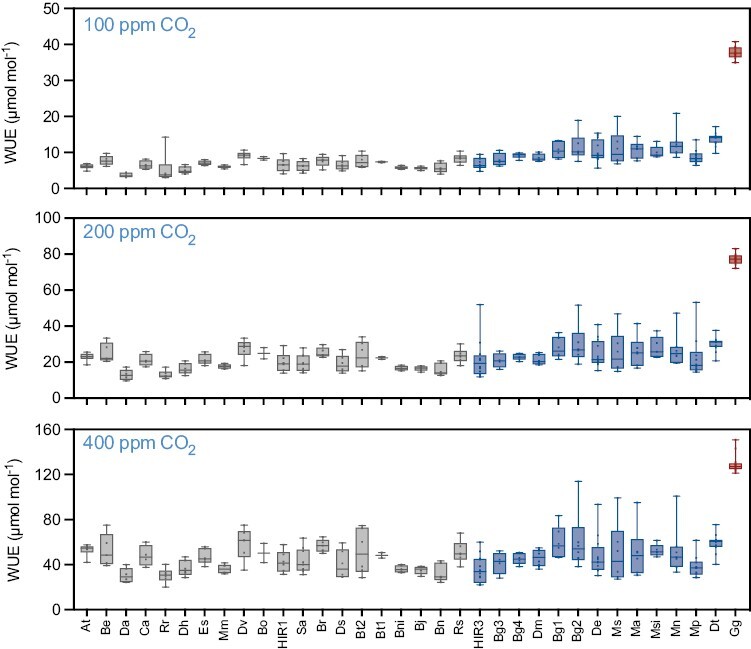
Water use efficiency (WUE) under different CO_2_ concentrations. WUE was calculated as the ratio between assimilation (*A*) and stomatal conductance (*g*_sw_). The gas exchange parameters were measured under conditions of ambient CO_2_ (400 ppm) or reduced CO_2_ concentrations of 200 ppm and 100 ppm. The tested taxa were sorted according to their CO_2_ compensation points and coloured according to the photosynthesis type as C_3_ (grey), C_3_–C_4_ (blue), and C_4_ (red). Taxa names have been abbreviated for legibility and are provided in Fig. 2 and the Materials and methods.

In addition to the above, operating a CO_2_-concentrating mechanism also yielded benefits in terms of improved WUE (ratio between CO_2_ assimilation and water stomatal conductance). For example, under ambient 400 ppm CO_2_, the WUE was significantly higher in the C_4_ species *G. gynandra* as compared with all other C_3_ and C_3_–C_4_ species (Fig. 4). On average, WUE did not differ between C_3_ and C_3_–C_4_ accessions at 400 ppm CO_2_. However, C_3_–C_4_ plants were found to exhibit a significantly improved WUE at both 200 ppm and 100 ppm CO_2_ compared with C_3_ species (*t*-test, *P*>0.05; Supplementary Fig. S1), recapitulating the trend observed for the assimilation rate. In addition, a strong negative correlation between WUE and *C*i was found across species at all atmospheric CO_2_ concentrations (Supplementary Fig. S2). Given that changes in stomatal conductance exhibited no photosynthesis type-specific pattern (Supplementary Fig. S1), this result suggests that higher WUE is achieved across species in the *Brassicaceae* by CO_2_ assimilation at lower internal *C*i, Thus, C_3_–C_4_ species are able to operate at lower internal CO_2_ concentrations than species from the C_3_ group.

Next, to determine the effect of C_3_, C_3_–C_4_, and C_4_ metabolism on the light reactions of photosynthesis, chlorophyll fluorescence parameters were also measured. In general, a positive correlation was found between net assimilation and ETR, as well as between assimilation rate and effective quantum efficiency *F*_v_ʹ/*F*_m_ʹ (Supplementary Fig. S2). However, fluorescence parameters were less affected under reduced atmospheric CO_2_ concentrations as compared with the assimilation rate (Supplementary Fig. S2).

**Fig. 5.**
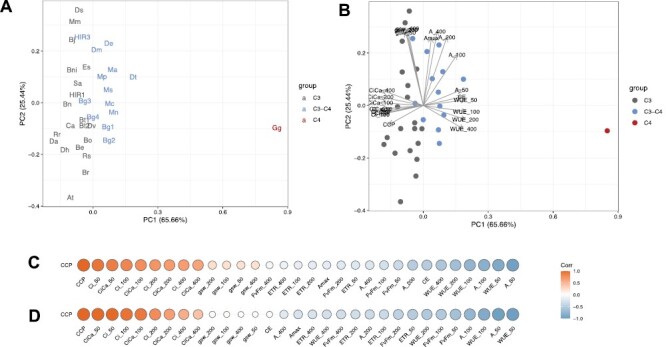
Principal component analysis (PCA) and correlations of the CO_2_ compensation point (CCP) with selected gas exchange parameters. Average values for the selected photosynthetic parameters determined under 50–400 ppm of CO_2_ were used for the analysis. (A) Localization of the plant lines in the PCA, (B) PCA including parameter loadings, (C) Pearson correlation coefficients demonstrated as heatmaps using all taxa, and (D) Pearson correlation coefficients demonstrated as heatmaps using only C_3_ and C_3_–C_4_ lines. The tested taxa were coloured according to photosynthesis types as C_3_ (grey), C_3_–C_4_ (blue), and C_4_ (red). Taxa names have been abbreviated for legibility and are provided in Fig. 2 and the Materials and methods. The dataset included CO_2_ compensation point (CCP), carboxylation efficiency (CE), assimilation (A), internal CO_2_ concentration (Ci), stomatal conductance (gsw), water use efficiency (WUE), ratio of internal to external CO_2_ concentrations (CiCa), electron transport rate (ETR), and quantum efficiency *F*_v_*ʹ*/*F*_m_ʹ (FvFm). The numbers after the parameter abbreviation indicate the CO_2_ concentration in the outside the leaf in the measuring cuvette.

To further assess how different photosynthesis types are characterized by differences in leaf physiological parameters, a principal component analysis (PCA) was performed. Since the importance of carbon-concentrating mechanisms becomes more obvious when CO_2_ is limited, gas exchange measurements at CO_2_ concentrations of 200, 100, and 50 ppm were included in this analysis in addition to those measured under ambient 400 ppm CO_2_ (Fig. 5A, B). In this PCA, the first principal component was found to explain 65.5% of the variation, and separates the C_4_ species *G. gynandra* from all other C_3_ and C_3_–C_4_ plants. To a lesser extent, the same component also separates C_3_ and C_3_–C_4_ plants, though these groups do overlap on this axis (Fig. 5A, B). As expected from the above results, the first principal component is driven by WUE, *C*i, *C*i/*C*a ratio, CCP, and CE. In contrast, the second principal component, driven by stomatal conductance and assimilation at higher CO_2_ concentrations, has no effect on separating plants across different photosynthesis types and is driven by species-specific variation.

As CCP was used above to classify different photosynthesis types, correlations were investigated between CCP values across species and all other measured leaf physiological parameters. A strong positive correlation was observed between CCP and both *C*i and the *C*i/*C*a ratio at low CO_2_ concentrations, respectively (Fig. 5C, D). In contrast, a negative correlation was found between CCP and both WUE and assimilation at low CO_2_ (Fig. 5C, D). These relationships were independently observed irrespective of whether the C_4_ species *G. gynandra* was included in the analysis or not. Conversely, CE (=initial slope of the *A*–*C*i curve) was negatively correlated with CCP only when the C_4_*G. gynandra* was included in the analysis (Fig. 5C, D). This indicates that CE was not influenced considerably during the transition from C_3_ to C_3_–C_4_, but only during transition from C_3_–C_4_ to C_4_.

### Metabolite profiles of leaves with different photosynthesis pathways

**Fig. 6.**
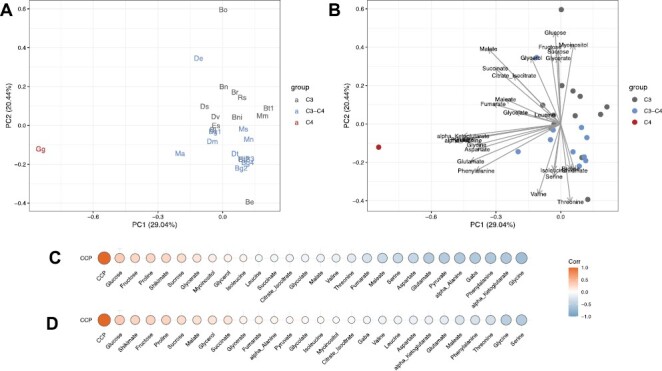
Principal component analysis (PCA) and correlations of the CO_2_ compensation point (CCP) with specific metabolites. Average values for the selected metabolites per taxon were used for the analysis. (A) Localization of the plant lines in the PCA, (B) PCA including metabolite loadings, (C) Pearson correlation coefficients demonstrated as heatmaps using all plant lines, and (D) Pearson correlation coefficients demonstrated as heatmaps using only C_3_ and C_3_–C_4_ lines. The tested taxa were coloured according to photosynthesis type as C_3_ (grey), C_3_–C_4_ (blue), and C_4_ (red). Taxa names have been abbreviated for legibility and are provided in Fig. 2 and the Materials and methods.

To assess the identity of potential transport metabolites used in C_3_–C_4_ intermediates, leaf primary metabolites of sampled species were also quantified and analysed by PCA (Fig. 6A, B). In this analysis, the first principal component explained 28.25% of variation and distinguishes C_3_/C_3_–C_4_ and C_4_ leaf biochemistry. Mainly responsible for this separation are high levels of α-alanine, α-ketoglutarate, aspartate, glycine, glutamate, pyruvate, phenylalanine, and γ-aminobutyric acid (GABA) in the C_4_*G. gynandra* compared with the C_3_ and C_3_–C_4_ background (Fig. 6B; Supplementary Table S3). In contrast, the second principal component sorts the majority of C_3_ species (clustered to the top of this axis) from C_3_–C_4_ species (clustered to the bottom of this axis) (Fig. 6A, B). Here, on this axis, the C_3_–C_4_ plants tend to have higher levels of serine, branched amino acids, and proline, whilst the C_3_ species are characterized by higher levels of glucose, sucrose, and myo-inositol.

**Fig. 7.**
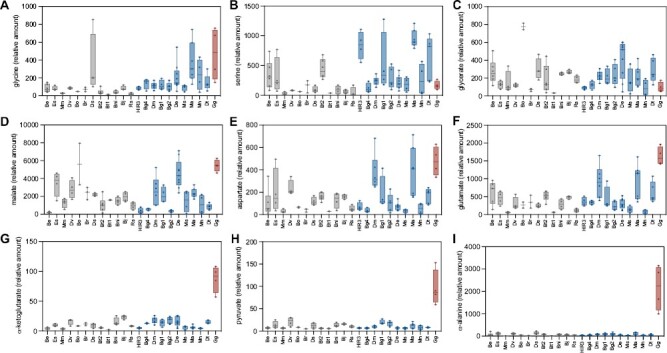
Metabolites in mature leaves of selected *Brassicaceae*. Relative amounts of glycine (A), serine (B), glycerate (C), malate (D), aspartate (E), glutamate (F), α-ketoglutarate (G),and pyruvate and α-alanine in selected plant accessions. The tested taxa were sorted according to their CO_2_ compensation points and coloured according to photosynthesis type as C_3_ (grey), C_3_–C_4_ (blue), and C_4_ (red). Taxa names have been abbreviated for legibility and are provided in Fig. 2 and the Materials and methods.

Correlation analyses of CCP values with primary metabolite levels were also performed across species (Fig. 6C, D; Supplementary Fig. S3). A negative correlation was observed between CCP and the C_4_-related metabolites α-alanine, α-ketoglutarate, aspartate, glutamate, and pyruvate (Figs 6, 7; Supplementary Table S3). However, this relationship seems to be driven by the strong accumulation of these metabolites in the C_4_ species alone (Fig. 6C). To identify metabolites that are potentially specific to C_3_–C_4_ photosynthesis, the correlation analysis was repeated without the C_4_ outgroup species. This resulted in the reduction of the strength of all statistical associations. Specifically, only serine and glycine showed significant negative correlations with CCP (Fig. 6D; Supplementary Table S3). Thus, this suggests that serine and glycine have a ubiquitous role in the glycine shuttle across all C_3_–C_4_ intermediates in the *Brassiceae*. Interestingly, however, glycine was the only metabolite that increased between C_3_ and C_3_–C_4_ species which was also high in the C_4_ species (Fig. 7A; Supplementary Figs S3, S4). In contrast, serine was enhanced among C_3_–C_4_ species compared with C_3_ species, but was detected at a C_3_ level in the C_4_*G. gynandra* (Fig. 7B; Supplementary Figs S3, S4). In the present study, glutamate, α-alanine, aspartate, pyruvate, malate, and α-ketoglutarate formerly predicted to be involved in nitrogen shuttling of C_3_–C_4_ leaves (Mallmann *et al.*, 2014) were not associated with CCP (Figs 6D, 7). Instead, levels of these metabolites were high in only some, but not all, C_3_–C_4_ taxa. For instance, glutamate and aspartate levels were relatively high in *M. arvensis*, *D. muralis*, and *D. tenuifolia*, but not in the other C_3_–C_4_*Moricandia* species *M. nitens* and *M. suffruticosa* (Fig. 7). In contrast, *D. erucoides* separated from the majority of other C_3_–C_4_ species in the PCA (Fig. 6A, B), showing relatively high levels of glycerate, glycolate, and malate (Fig. 7; Supplementary Table S3). In summary, the present results describe a general role for only glycine and serine as predicted shuttle metabolites in C_3_–C_4_ biochemistry across all species.

### Association of CCP with structural features of the bundle sheath

**Fig. 8.**
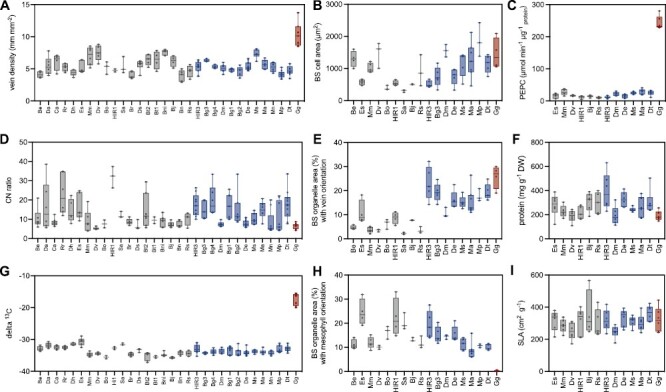
Leaf structure- and composition-related parameters and PEPC activity. Mature leaves were used for determination of vein density (A), bundle sheath cell area in micrographs (B), PEPC activity (C), carbon to nitrogen ratio (D), area of bundle sheath organelles with vein orientation in micrographs (E), protein content (F), ^13^C signature (G), area of bundle sheath organelles with orientation to intercellular space or mesophyll in micrographs (H), and specific leaf area (I). The tested taxa were sorted according to their CO_2_ compensation point and coloured according to photosynthesis type as C_3_ (grey), C_3_–C_4_ (blue), and C_4_ (red). Taxa names have been abbreviated for legibility and are provided in Fig. 2 and the Materials and methods.

Given that leaf and BS cell architecture play an important role in underpinning CO_2_-concentrating mechanisms by enabling adequate metabolite transport between M and BS tissue, we also sought to characterize the leaf anatomy of our *Brassicaceae* species. In the present study, it was observed that vein density was highest in the C_4_*G. gynandra* compared with all other species. However, no difference in vein density was observed between C_3_ and C_3_–C_4_ plant accessions (Fig. 8A; Supplementary Fig. S5). To determine whether differences in BS structure were present between photosynthetic types, a representative subset of plant accessions were studied in more detail by light microscopy (Supplementary Fig. S6). In this analysis, although BS cross-section area was high in the C_4_ species as well as in several C_3_–C_4_ species, it was not found to be significantly different between C_3_ and C_3_–C_4_ plants (Fig. 8B; Supplementary Fig. S7). Moreover, within the BS cells, the areas occupied by plastids and other organelles with either vein (inner half) or ICS/M orientation (outer half) were determined. Areas with ICS/M-oriented organelles did not differ between C_3_ and C_3_–C_4_ leaf cross-sections (Fig. 8E). In the C_4_ leaf, none of the BS organelles faced the outer ICS/M side. On the other hand, all plant accessions with a CCP <40 ppm featured enhanced organelle accumulation around the vein (Fig. 8H). This resulted in higher total organelle area in the C_3_–C_4_ BS cells. Thus, organelle abundance and orientation probably played a decisive role for the functioning of weak CO_2_-concentrating mechanisms.

C_4_ anatomy consists of just one layer of BS and M cells around the veins, which limits the total number of cell layers. In our study, the C_4_ leaves of *G. gynandra* were comparably thin. However, leaf thickness within the C_3_ and C_3_–C_4_ groups showed species-specific variation. For instance, independent of photosynthesis type, all *Moricandia* species possessed thick succulent leaves. Values of SLA are usually greater for C_4_ than for C_3_ leaves (Atkinson *et al.*, 2016), but no pronounced photosynthesis type-related differences in SLA could be observed in our study (Fig. 8I).

The C_4_ pathway allows plants to fix CO_2_ with lower nitrogen input. This means that typically, C_4_ plants have lower leaf nitrogen concentrations compared with C_3_ species (Long, 1999; Craine *et al.*, 2005; Gowik *et al.*, 2011). Interestingly, however, C_4_*G. gynandra* in our analysis had surprisingly high leaf nitrogen concentrations and low leaf CN ratios (Fig. 8D). This result could be influenced by the slow growth rate of this species in comparison with the majority of *Brassicaceae* species in this study. However, leaf protein concentrations in this C_4_*G. gynandra* were low relative to the background of other species, indicating C_4_-specific differences in nitrogen allocation (Fig. 8F). Interestingly, no difference in CN ratios or leaf protein concentrations could be observed between the C_3_ and C_3_–C_4_ species (Fig. 8D; Supplementary Fig. S7).

Operation of the C_4_ pathway required increased activity of PEPC, but allows reduction in concentrations of Rubisco and CBB cycle enzymes (Brautigam *et al.*, 2011; Gowik *et al.*, 2011). In our study, PEPC activity was 8- to 20-fold higher in the C_4_*G. gynandra* leaves as compared with the leaves of the C_3_ and C_3_–C_4_ species (Fig. 8C; Supplementary Table S4). PEPC activities varied in the individual plant taxa, but were not significantly different between the C_3_ and C_3_–C_4_ groups (Supplementary Fig. S7). *Eruca sativa* and *M. moricandioides*, in particular, showed PEPC activities similar to the C_3_–C_4_ taxa *M. arvensis*, *M. suffruticosa*, and *D. tenuifolia* (Fig. 8; Supplementary Fig. S7). These results emphasize the power of our multispecies analysis that allows distinction between species- and photosynthesis type-related variation.

**Fig. 9.**
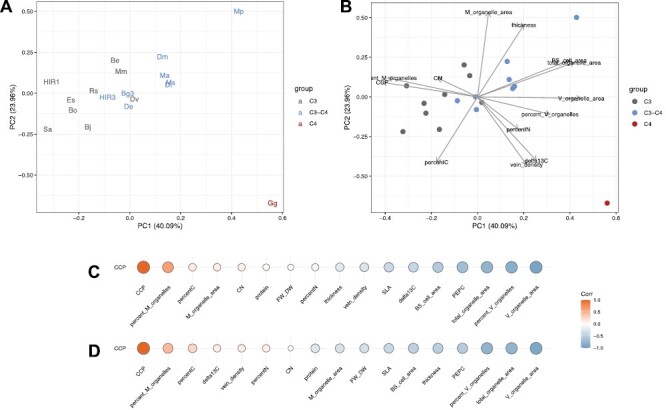
Principal component analysis (PCA) and correlation of the CO_2_ compensation point (CCP) with leaf structural and compositional components. Average values for the selected parameters measured by EA-IRMS analysis of leaf cross-sections by light microscopy. (A) Localization of the taxa in the PCA, (B) PCA including parameter loadings, (C) Pearson correlation coefficients demonstrated as heatmaps using all plant lines, (D) Pearson correlation coefficients demonstrated as heatmaps using only C_3_ and C_3_–C_4_ lines. The tested taxa were coloured according to photosynthesis type as C_3_ (grey), C_3_–C_4_ (blue), and C_4_ (red). Taxa names have been abbreviated for legibility and are provided in Fig. 2 and the Materials and methods.

Summarizing the above-mentioned structural and leaf composition-related parameters in a PCA, the C_4_*G. gynandra* can be separated from the rest of the *Brassicaceae* plants (Fig. 9A, B). This was mainly driven by high values for δ^13^C, vein density, and vein-orientated organelles in the BS as well as low values for CCP and ICS/M-orientated organelles in the BS (Fig. 9A, B). C_3_ and C_3_–C_4_ accessions separated along the same line in a combination of PC1 and PC2, but an overlap between the two groups was nevertheless observed. Correlation of the CCP to the selected components supported the importance of organelle accumulation and orientation in the BS for the activity of the C_4_ as well as the C_3_–C_4_ pathway (Fig. 9C, D; Supplementary Fig. S8).

## Discussion

### Physiological and phylogenetic analysis indicate evolution of multiple independent C _3_ –C _4_ lineages in the *Brassiceae*

Our survey revealed multiple origins of C_3_–C_4_ photosynthesis in the *Brassiceae* tribe (Figs 1, 2), ranging from very efficient photorespiratory shuttles in *D. tenuifolia* and the *Moricandia* genus (*M. suffruticosa*, *M. arvensis*, *M. sinaica*, *M. nitens*, and *M. spinosa*), to relatively weaker carbon-concentrating mechanisms in *B. gravinae*, *D. erucoides*, and *H. incana* HIR3. The carbon-concentrating mechanism in *D. muralis* is assumed to be inherited from the C_3_–C_4_ parent *D. tenuifolia* during natural hybridization with the C_3_ species *D. viminea* (Eschmann-Grupe *et al.*, 2003; Ueno *et al.*, 2006).

Interestingly, from the two taxa assigned as *H. incana*, only one (HIR3) showed C_3_–C_4_-like features such as a CCP below 40 ppm and the typical organelle arrangement in the BS cells. Comparison of chloroplast sequences from both accessions revealed that only HIR1 clustered together with other accessions of this species while HIR3 sequences clustered closer to *Sinapis pubescencs* and *Brassica procumbens* (Guerreiro *et al.*, 2023). This suggests that HIR3 and *H. incana* belong to different species, and the former represents a new C_3_–C_4_ lineage in the *Brassiceae*.

### *Brassicaceae* display large variation in efficiency of the carbon conservation mechanism but no C _4_ -like shuttles

Our survey of CO_2_-concentrating mechanisms in the *Brassicaceae* confirmed that measurements of the CCP represent a valuable tool for the identification of C_3_–C_4_ intermediate plant accessions. In agreement with the large CCP screening study by Krenzer *et al.* (1975) and our own statistical analysis, taxa with CCPs between 10 ppm and 40 ppm were classified as C_3_–C_4_ intermediates. In this group, we observed gradual changes in the CO_2_-concentrating capacity. Our study therefore supports models claiming that after establishment of the basic photorespiratory shuttle, multiple metabolic and anatomic adjustments can contribute to the efficiency of the pathway, resulting in additive small fitness gains (Heckmann *et al.*, 2013).

The lowest CCPs in the present investigation were measured in *D. tenuifolia* and the C_3_–C_4_*Moricandia* species. Although various accessions of these species were used in different studies (Hylton *et al.*, 1988; Rawsthorne *et al.*, 1988a; Apel *et al.*, 1997; Ueno *et al.*, 2003, 2006; Schlüter *et al*., 2017), low CCPs seem to be a ubiquitous trait of these respective species. Moreover, low CCPs in these species were supported by BS-specific localization of the GLDP protein (Hylton *et al.*, 1988; Rawsthorne *et al.*, 1988a; Ueno *et al.*, 2003). Further, especially in *D. tenuifolia*, CCP values were observed as very low and close to those typical of C_4_ species. However, the strict separation of the C_4_*G. gynandra* in all PCAs and especially the PEPC and ^13^C measurements support previous observations claiming absence of C_4_-like shuttles in the *Brassicaceae* (Holaday and Chollet, 1984; Hunt *et al.*, 1987; Sage *et al*., 2011).

### Reduction in CCP correlates negatively with organelle accumulation and arrangement in the BS

Despite the differences in efficiency of the photorespiratory shuttle, changes in organelle arrangement were observed in all taxa classified as C_3_–C_4_. For instance, all C_3_–C_4_ taxa possessed an enhanced BS area occupied by organelles in the centripetal position and a higher total organelle area per BS cell compared with C_3_ species (Figs 8, 9). This underlines the importance of anatomical features for carbon-recapturing mechanisms. A strong correlation between reduction in CCP and increased organelle accumulation facing the vein in the BS was also previously observed in interspecific hybrids between *D. tenuifolia* (C_3_–C_4_) and *R. sativus* (C_3_) (Ueno *et al.*, 2003). The BS structural features appeared to be genetically encoded and are inherited independently from the GLDP localization (Ueno *et al.*, 2003). Residual expression of *GLDP* was also observed in M cells of C_3_–C_4_ intermediate *Flaveria* species (Schulze *et al.*, 2013). This shows that structural modifications can underpin an effective CCP without complete suppression of GLDP in the M cells.

C_3_–C_4_ intermediates in our study contained several layers of M cells such that many do not directly border BS cells. So complete absence of GDC activity in the M cells would require transport of photorespiratory glycine through other M cell layers prior to entering the BS for metabolization. However, accumulation of mitochondria in the BS might create a glycine sink supporting glycine diffusion to the BS, and partial reduction of M GLDP expression would enforce the shuttle. TEM studies of centripetally localized organelles from C_3_–C_4_*Brassicea*e (Ueno *et al.*, 2006; Schlüter *et al*., 2017), *Asteraceae* (McKown and Dengler, 2007), *Boraginaceae* (Muhaidat *et al.*, 2011), *Scrophulariaceae* (Khoshravesh *et al.*, 2012), *Arthropogoninae* (Khoshravesh *et al.*, 2016), and *Chenopodiaceae* (Yorimitsu *et al.*, 2019) have shown a close arrangement of mitochondria and chloroplasts. Thus, BS ultrastructure seems to play a major role in prevention of photorespiratory CO_2_ and NH_3_ loss and in improvement of leaf carbon and possibly also nitrogen economy.

In contrast to the C_4_ species in our study, BS cells in C_3_–C_4_*Brassicaceae* exhibited organelles facing the ICS and M cells (Fig. 8; Supplementary Fig. S6). This amount of ICS/M cell-facing organelles decreased in C_3_–C_4_ species with higher carbon-concentrating efficiency. Our results suggest that accumulation of centripetal organelles and reduction of peripheral organelles are not necessarily regulated by the same process. Additional structural features of C_4_ species such as enlarged BS cell area and higher vein density did not differ between the tested C_3_ and C_3_–C_4_*Brassicaceae* taxa. Further, leaf thickness, SLA, and FW/DW ratios were also not different between the leaves of the C_3_, C_3_–C_4_, and C_4_ taxa (Fig. 8; Supplementary Fig. S8). Thus, despite leaf anatomy and BS architecture being important requirements for evolution of carbon-concentrating mechanisms (Christin *et al.*, 2013), modifications in leaf succulence parameters do not appear to be essential for an efficient photorespiratory carbon-concentrating pathway. Plasticity in some morphological parameters could also play a role in further evolution towards the C_4_ leaf, and it could be speculated that limited genetic potential for the adjustment of vein density and mesophyll structure could be connected to the absence of C_4_-like shuttles in the *Brassicaceae*.

### *Brassicaceae* C _3_ –C _4_ metabolism had only a minor influence on leaf steady-state metabolite patterns

Beside organelle arrangement in the BS, the shift of GDC activity to this tissue influences leaf biochemistry (Rawsthorne, 1992; Schlüter *et al*., 2017). Relocation of the GLDP protein to the BS has been observed in all investigated C_3_–C_4_ classified species to date (Schlüter and Weber, 2016) and is therefore seen as the decisive step for the evolution of a photorespiratory carbon concentration shuttle. In the *Brassicaceae*, BS specificity of GLDP was shown for different *Moricandia* species (Rawsthorne *et al*., 1988a), *Diplotaxis tenuifolia* (Ueno *et al.*, 2003), and *Brassica gravinae* (Ueno, 2011). Lack or reduction of GDC activity in the M causes accumulation of photorespiratory glycine and its transport along the concentration gradient to the BS. The GDC reaction converts two molecules of glycine into one molecule of serine, but also utilizes NADH and releases NH_3_ alongside CO_2_. This imbalance requires further metabolic readjustment between the two cell types. Nevertheless, beyond GLDP localization, not much is known about the cell-specific metabolism or the nature of additional metabolite shuttles in C_3_–C_4_*Brassicaceae*.

If metabolite exchange between M and BS cells is realized by a concentration gradient, high concentrations of these transported metabolites would be expected in the leaves (Leegood and von Caemmerer, 1989). However, it should be noted that high metabolic flux and cell-specific metabolite accumulation might mask these gradients in total leaf extracts. Our metabolite analysis did not identify preferential metabolite shuttles operating across all C_3_–C_4_*Brassicaceae* species. Steady-state glycine concentrations were generally enhanced in the C_3_–C_4_ species compared with C_3_ species, supporting the hypothesis that glycine is transported from the M cells to the BS for decarboxylation. High glycine was, however, also found in leaves of the C_3_*D. tenuisiliqua* and the C_4_ species *G. gynandra*, indicating that glycine accumulation is not a distinct C_3_–C_4_ feature (Fig. 7A). Further uncertainty exists around the metabolites transported back from the BS to the M for rebalancing of carbon, nitrogen, and energy metabolism (Borghi *et al.*, 2022). Beside glycine, serine accumulation also exhibited a negative correlation with CCP values (Fig. 6D). This strongly supports the involvement of serine as a metabolite transported back from the BS to the M cells (Rawsthorne, 1992; Mallmann *et al.*, 2014), although variation in serine levels suggests that the contribution of serine transport could vary between the different taxa.

High variation between the individual taxa also existed for other shuttle metabolite candidates. Modelling approaches have previously predicted the involvement of glutamate, α-ketoglutarate, alanine, pyruvate, aspartate, and malate in shuttling processes for rebalancing of nitrogen metabolism between the M and BS (Mallmann *et al.*, 2014). Malate and aspartate could also be involved in rebalancing of reducing power between the two cell types (Johnson *et al.*, 2021). Contributions of glutamine/glutamate and asparagine/aspartate to intercellular shuttles were suggested for the C_3_–C_4_ species *Flaveria anomala* (Borghi *et al.*, 2022). Here, enhanced levels of these various metabolites could be observed in some, but not all, C_3_–C_4_ taxa (Fig. 7). For example, high concentrations of malate, aspartate, and glutamate were found in species displaying very low CCPs such as *M. arvensis* and *D. tenuifolia*. Interestingly, the C_3_–C_4_*Moricandia* species which supposedly share a single C_3_–C_4_ evolutionary origin also showed strong variation in the metabolite pattern. A similar absence of main shuttle metabolites has also been described for C_3_–C_4_*Flaveria* species (Borghi *et al.*, 2022). Our data generally support the hypothesis that multiple metabolites are transported between the M and BS (Schlüter *et al*., 2017; Borghi *et al.*, 2022). The contribution of the different metabolites could differ in the individual taxa depending on genetic as well as environmental influences. Such a multitude of solutions indicates that metabolite and energy balancing does not represent a limiting step during evolution of carbon-concentrating pathways.

To date, enzyme localization studies have mostly focused on the GLDP protein, and much less is known about whether other reactions are shifted to the BS in C_3_–C_4_ species. In *M. arvensis*, other tested photorespiratory enzymes such as glycolate oxidase, serine hydroxymethyl transferase, and other subunits of the GDC complex were present in both cell types (Morgan *et al.*, 1993). Enzyme activities in *M. arvensis* in M- and BS-enriched fractions were also equally distributed for glyoxylate aminotransferases, glycolate oxidase, and hydroxypyruvate reductase (Rawsthorne *et al.*, 1988b), supporting the crucial role of GLDP for uneven distribution for glycine shuttle operation in *M. arvensis*. On the other hand, all GDC subunits were preferentially expressed in the BS in C_3_–C_4_*Flaveria* and *Panicum* species (Morgan *et al.*, 1993). Shifting of additional photorespiratory steps could considerably influence the metabolite shuttles. In our study, some species, especially *D. erucoides* and *D. tenuifolia*, showed high levels of glycolate and glycerate. Interestingly, intercellular transport of glycerate and glycolate was predicted in a constraint-based modelling approach for weak carbon-concentrating mechanisms on the evolutionary path to C_4_ photosynthesis (Blätke and Bräutigam, 2019). Exchange of these metabolites between M and BS would reduce the need for intercellular nitrogen recycling (Borghi *et al.*, 2022). Part of the photorespiratory metabolites could also feed into additional pathways in the BS. It has been estimated that 1–5% of the photorespiratory glycine and ~30% of serine can be metabolized outside the photorespiratory cycle in processes such as protein biosynthesis (Busch *et al.*, 2018). The high organelle accumulation would increase the demand for protein synthesis in the C_3_–C_4_ BS. Furthermore, the BS is also responsible for loading of assimilation products into the phloem, and part of the carbon and nitrogen transported into the BS by the glycine shuttle could support metabolite export to the sink tissue of the plants.

### C _3_ –C _4_ photosynthesis is associated with reduced *C*i and enhanced WUE especially under limiting CO _2_

In the *Brassicaceae*, the presence of C_3_–C_4_ metabolism did not translate into improved photosynthetic assimilation under ambient environmental conditions (Figs 3, 4). For instance, across the *Brassicaceae* species analysed in the current study, assimilation rates appeared to be genotype specific rather than related to photosynthesis type under ambient CO_2_. This lack of correlation between assimilation and photosynthesis type has also been previously described in the *Chenopodiaceae* (Yorimitsu *et al.*, 2019).

Interestingly, however, C_3_–C_4_ taxa in the *Brassicaceae* adjusted leaf *C*i to lower levels compared with C_3_ taxa in this clade. The difference between these photosynthesis types was marginal under ambient conditions, but became more pronounced under CO_2_ conditions of ≤200 ppm (Fig. 3). The ability to assimilate CO_2_ at lower *C*i translated into higher WUE in the C_3_–C_4_ taxa compared with the C_3_ species. This increase in WUE observed was underpinned by enhanced assimilation, as stomatal conductance was similar among the C_3_ and C_3_–C_4_ taxa under all tested conditions (Fig. 4). It should be noted, however, that the differences observed for *C*i and WUE between C_3_ and C_3_–C_4_ taxa were small in comparison with the difference between all C_3_ and C_3_–C_4_ taxa and C_4_*G. gynandra*, thus underlining the superiority of the C_4_ pathway as a CO_2_-concentrating mechanism compared with C_3_–C_4_ metabolism. Similar observations have been previously made in *Heliotropium* and *Flaveria*, in which C_3_–C_4_ species achieved WUE values between those of the C_3_ and C_4_ species. This was also due to higher assimilation rather than modified conductance (Huxman and Monson, 2003; Vogan *et al.*, 2007). These results support an advantage of the C_3_–C_4_ pathway in high photorespiratory conditions which cause CO_2_ restriction due to stomatal closure.

Evolution of the glycine shuttle often appears to be connected to an enlargement of the growth habitat (Lundgren and Christin, 2017). The C_3_ species *M. moricandioides* for instance seems to be geographically restricted to the Iberian Peninsula, while the closely related C_3_–C_4_ species *M. arvensis* has spread into northwest Africa, Southern Europe, and other parts of the planet where it is mostly associated with cultivated areas and disturbed sites (Perfectti *et al.*, 2017). *Diplotaxis tenuifolia* also often grows as an invasive species occupying sunny, harsh, and arid areas (Nicoletti *et al.*, 2007) in which water, nutrient, and temperature conditions can change rapidly. It is therefore possible that C_3_–C_4_ species profit from high environmental plasticity of the trait.

Ecological studies which have investigated the adaptation of C_3_–C_4_ species to specific environmental conditions are unfortunately still rare (Oono *et al.*, 2022). In contrast to C_3_ and C_4_ species, the C_3_–C_4_ compensation points are strongly influenced by environmental conditions, especially light, temperature, and nitrogen (Brown and Morgan, 1980; Holaday and Chollet, 1983; Hunt *et al.*, 1987; Schuster and Monson, 1990; Gomez *et al.*, 2020; Oono *et al.*, 2022). In *Chenopodium album*, the CCP was lowest under high temperature and low nitrogen conditions, which was connected to accumulation of the GLDP protein preferentially in the BS (Oono *et al.*, 2022). *Moricandia arvensis* leaves also had lower CCPs and higher WUE under hotter and more arid summer conditions than in milder spring climates (Gomez *et al.*, 2020). Plasticity of photosynthetic traits under stress conditions was recently also reported for the C_2_ species *Sasola divaricate* (Tefarikis *et al.*, 2022). Our results indicate that gradual and even facultative implementation of carbon shuttles between the M and BS are possible and should be considered in future experiments.

Knowledge about the distribution of species with glycine shuttle metabolism is generally still limited to studies among relatives of C_4_ species. This is mainly due to the dependence on gas exchange equipment and time-consuming measurements. It is therefore assumed that the frequency of species with weaker carbon-concentrating mechanisms is greatly underestimated (Sage *et al*., 2011; Lundgren, 2020). Identification of C_3_–C_4_ features in a *H. incana* HIR3 and recently also in some *C. album* accessions (Yorimitsu *et al.*, 2019) supports this hypothesis. As such, faster methods for identification of C_3–_C_4_ intermediates could help to close this knowledge gap. Here, our correlation analysis showed that measurements of assimilation at low CO_2_ are sufficient for detection of C_3_–C_4_ phenotype and would save considerable time as opposed to having to calculate CCP by measuring assimilation across a range of CO_2_ concentrations (Fig. 5D). For example, a very strong positive correlation in the present results was found to exist between CCP and assimilation rate at 50 ppm CO_2_, which is close to the CCP of C_3_ species. High and significant negative correlation to CCP also existed for WUE under CO_2_ conditions of ≤200 ppm. As in our experiments assimilation generally correlated positively with photosynthesis efficiency *F*_v_ʹ/*F*_m_ʹ, fluorescence combined with stomatal conductance measurements could possibly also be used in a fast initial screening experiments for identification of C_3_–C_4_ intermediates in the future.

### Conclusions

Our survey revealed that photorespiratory shuttles evolved up to five times in the *Brassiceae* tribe in different genetic backgrounds. Measurements of the CCP indicated considerable variation in the pathway in the different tested taxa. Reduction in CCP was generally associated with organelle arrangement in the BS. Thus, elucidation of regulatory mechanisms underlying organelle multiplication and arrangement in the BS appear to be crucial for engineering an efficient glycine shuttle pathway into the leaf.

Although CCPs as low as 12 ppm were observed in *D. tenuifolia*, there was no evidence for the operation of C_4_-like shuttles in the tested taxa, supporting its classification as a distinct pathway. All C_3_–C_4_ classified taxa belong to the *Brassiceae* tribe which appears to have lost one GLDP gene copy, suggesting that this event facilitated evolution of the glycine shuttle (Schlüter *et al*., 2017). Additional loss-of-function mutations or insertion of a transposable element are thought to be involved in loss or reduction of GDC activity in the M cells (Rawsthorne, 1992; Sage *et al*., 2012; Adwy *et al.*, 2015; Triesch *et al.*, 2023, Preprint). In *D. muralis*, transfer of weak carbon-concentrating mechanisms seem to have been inherited during hybridization from a C_3_–C_4_ parent (Ueno *et al.*, 2003). The contribution of hybridization to distribution of carbon-concentrating pathways has been discussed for several plant groups including *Sasola* and *Flaveria* (Kadereit *et al.*, 2017; Tefarikis *et al.*, 2022; Morales-Briones and Kadereit, 2023, Preprint). In some grasses, lateral gene transfer has been shown to support the rapid and successful establishment of the C_4_ pathway (Dunning *et al.*, 2019b). Such scenarios would nevertheless require donor species that are able to successfully transfer essential features into the receiving genetic background.

Our results reveal that photorespiratory carbon-concentrating mechanisms in the *Brassiceae* show large variation in their biochemical and physiological features. C_3_–C_4_*Brassiceae* species are often associated with fast changing temperature, water, and nutrient conditions. Metabolic plasticity could also be advantageous in crop species challenged by dynamic climatic variability. *Brassica napus* or *B. oleaceae* are closely related to the described C_3_–C_4_ species and would be prime targets for transfer of this trait. Recent progress in sequencing the genomes of these species and related species in the *Brassicaceae* (Guerreiro *et al.*, 2023) can help to identify the molecular mechanisms behind BS-specific C_3_–C_4_ architecture and biochemistry.

## Supplementary data

The following supplementary data are available at *JXB* online.

Table S1. Origin of the seed material.

Table S2. Physiological data measured with the Li-COR 6800 (average per accession, standard deviation, HSD group).

Table S3. Metabolite data from GC-MS measurements (average per accession, standard deviation, HSD group).

Table S4. Data from light microscopy, EA-IRMS, and PEPC enzyme assay (average per accession, standard deviation, HSD group).

Fig. S1. Box and whisker plot for gas exchange and fluorescence data summarized per photosynthesis type.

Fig. S2. Heatmap of Pearson correlation matrix for physiological gas exchange and fluorescence data.

Fig. S3. Heatmap of Pearson correlation matrix for CO_2_ compensation points and metabolite data from GC-MS analysis.

Fig. S4. Box and whisker plot for metabolite data summarized per photosynthesis type.

Fig. S5. Vein pattern in de-stained leaves.

Fig. S6. Micrographs of bundle sheath cross-sections.

Fig. S7. Box and whisker plot for structural and leaf composition data summarized per photosynthesis type.

Fig. S8. Heatmap of Pearson correlation matrix for CO_2_ compensation point, structural parameters from light microscopy analysis, leaf composition data from EA-IRMS analysis. and PEPC enzyme activity.

erad250_Suppl_Supplementary_Figures_S1-S8

erad250_Suppl_Supplementary_Tables_S1-S4

## Data Availability

The raw SRA data used for the phylogenetic tree are deposited in NCBI under BioProject PRJNA905373. Physiological, biochemical and anatomical data per taxon used in this study are available in Supplementary Tables S2–S4.
