# Supplementary material for: Brassicaceae display variation in efficiency of photorespiratory carbon-recapturing mechanisms
Source: J Exp Bot. 2023 Jul 1;74(21):6631–49. doi: 10.1093/jxb/erad250 (PMC10662225; doi:10.1093/jxb/erad250)
Supplement: erad250_Suppl_Supplementary_Figures_S1-S8 [file erad250_suppl_supplementary_figures_s1-s8.pdf]

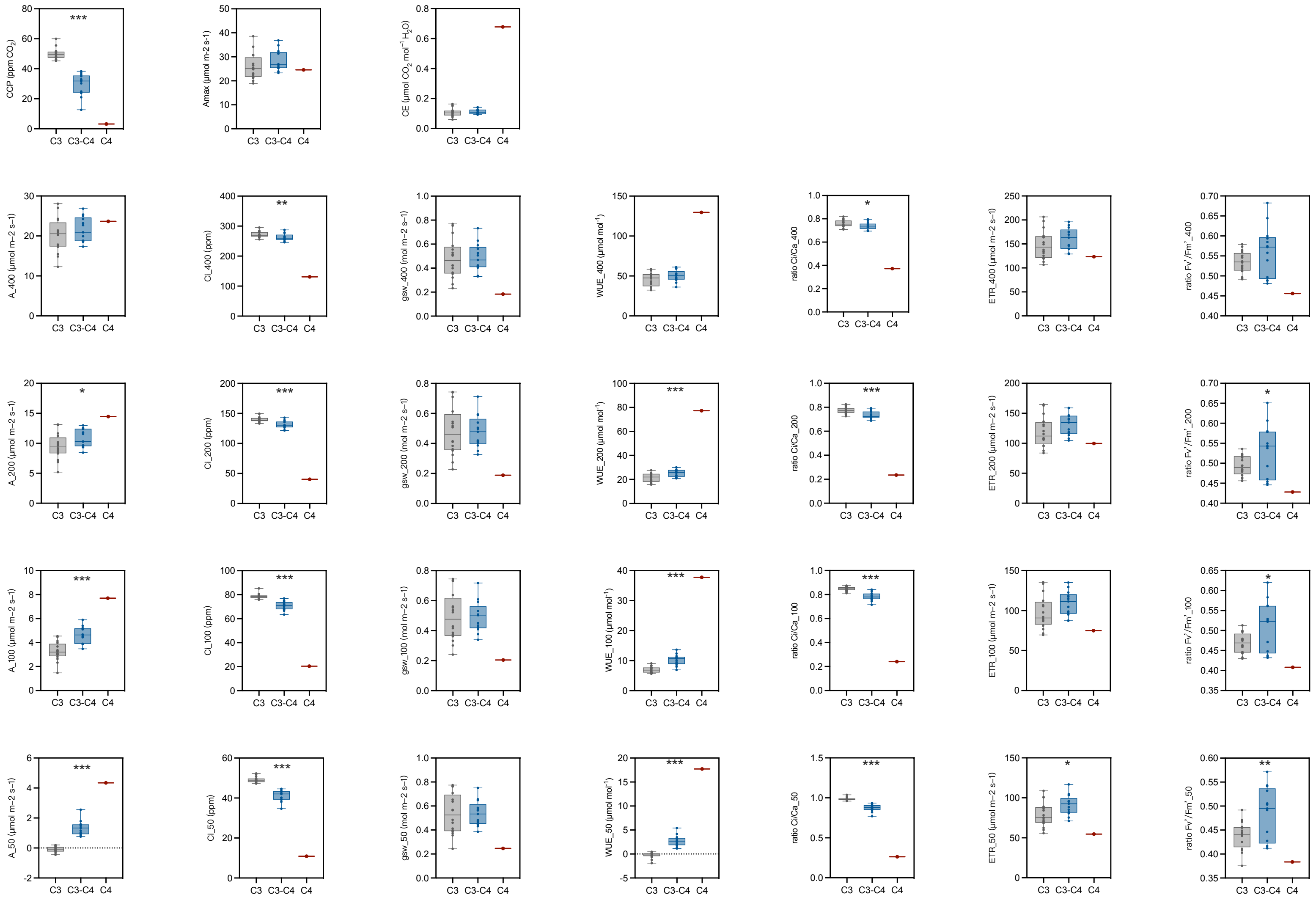

Supplemental Figure S1: Gas exchange and Fluorescence parameter sorted by photosynthesis group.
Significant differences between data for the C_3 and C_3-C_4 groups are indicated with stars (*** -> p<0.001; ** -> p<0.01; * -> p<0.05). The C_4 group is represented only by one line from the Cleomaceae (*G. gynandra*).

A

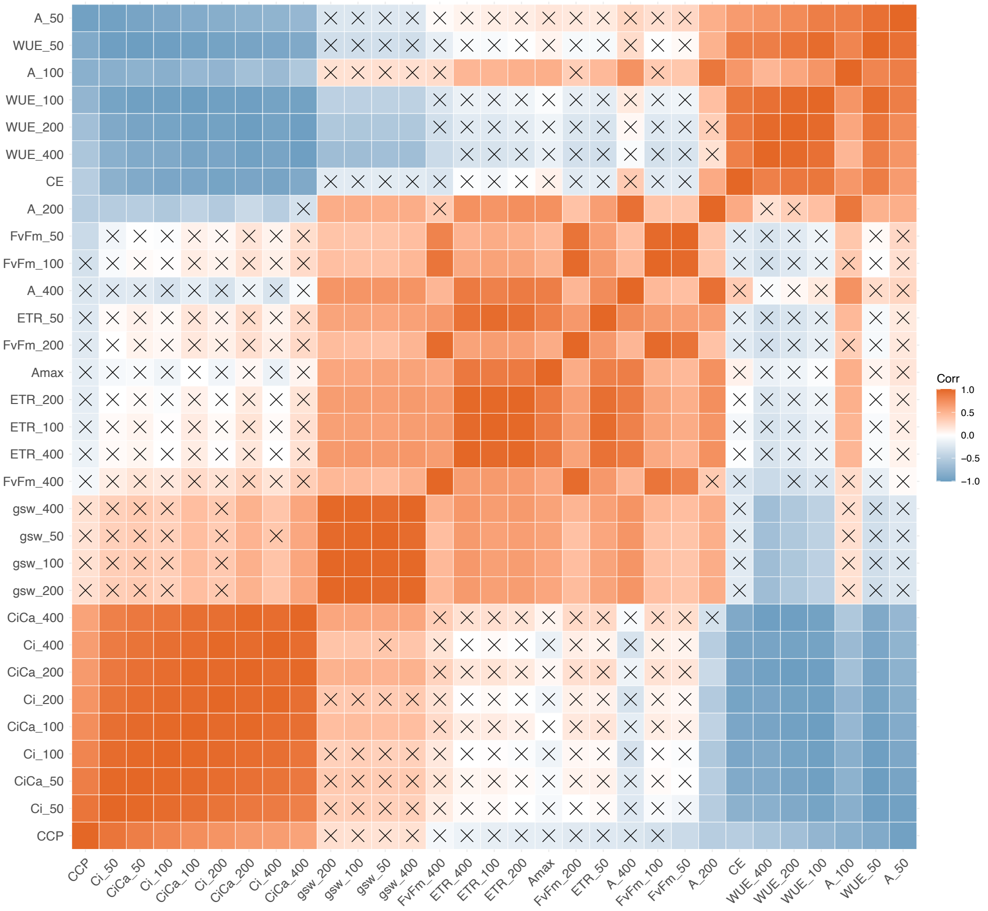
A_50
WUE_50
A_100
WUE_100
WUE_200
WUE_400
CE
A_200
FvFm_50
FvFm_100
A_400
ETR_50
FvFm_200
Amax
ETR_200
ETR_100
ETR_400
FvFm_400
gsw_400
gsw_50
gsw_100
gsw_200
CiCa_400
Ci_400
CiCa_200
Ci_200
CiCa_100
Ci_100
CiCa_50
Ci_50
CCP
CCP
Ci_50
CiCa_50
Ci_100
CiCa_100
Ci_200
CiCa_200
Ci_400
CiCa_400
gsw_200
gsw_100
gsw_50
gsw_400
FvFm_400
ETR_400
ETR_100
ETR_200
Amax
FvFm_200
ETR_50
A_400
FvFm_100
FvFm_50
A_200
CE
WUE_400
WUE_200
WUE_100
A_100
WUE_50
A_50
Corr
1.0
0.5
0.0
−0.5
−1.0

B

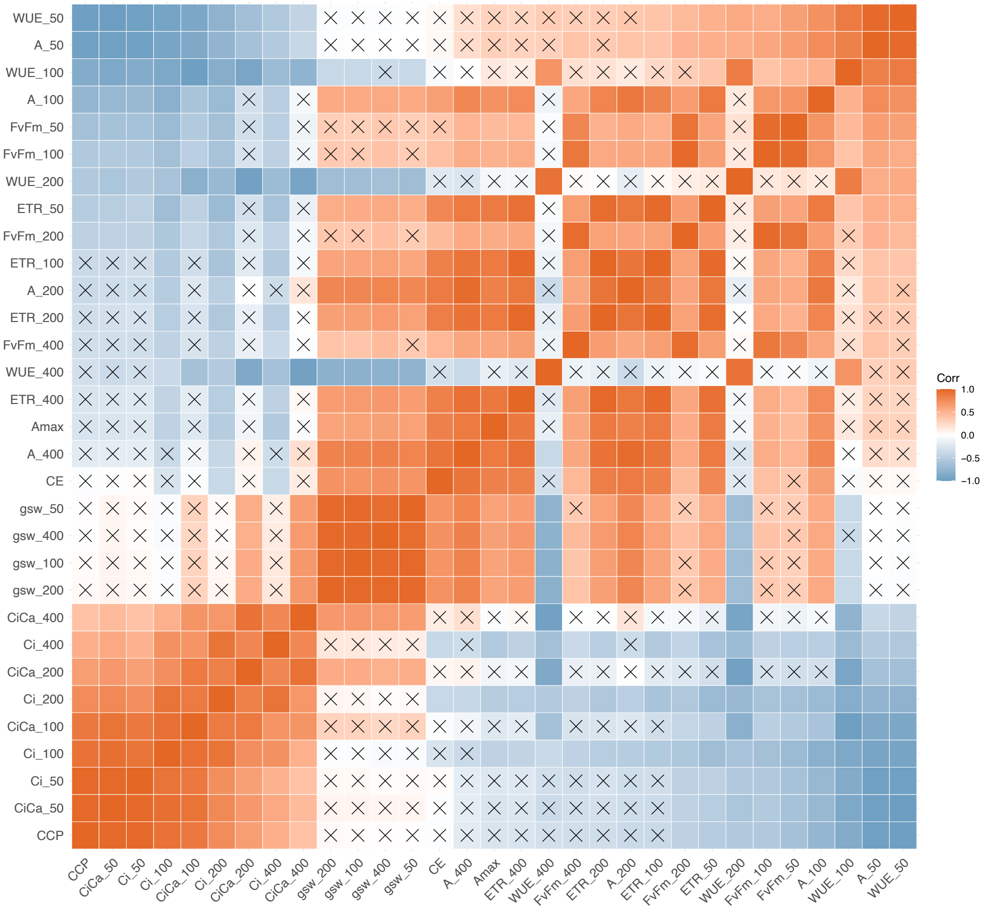
WUE_50
A_50
WUE_100
A_100
FvFm_50
FvFm_100
WUE_200
ETR_50
FvFm_200
ETR_100
A_200
ETR_200
FvFm_400
WUE_400
ETR_400
Amax
A_400
CE
gsw_50
gsw_400
gsw_100
gsw_200
CiCa_400
Ci_400
CiCa_200
Ci_200
CiCa_100
Ci_100
Ci_50
CiCa_50
CCP
CCP
CiCa_50
Ci_50
Ci_100
CiCa_100
Ci_200
CiCa_200
Ci_400
CiCa_400
gsw_200
gsw_100
gsw_400
gsw_50
CE
A_400
Amax
ETR_400
WUE_400
FvFm_400
ETR_200
A_200
ETR_100
FvFm_200
ETR_50
WUE_200
FvFm_100
FvFm_50
A_100
WUE_100
A_50
WUE_50
Corr
1.0
0.5
0.0
−0.5
−1.0

Supplemental Figure S2. Correlation matrix of photosynthetic parameters
Photosynthetic gas exchange and fluorescence parameter were determined using a LICOR 6800 during a CO_2 response curve including parameter measured at 400, 200, 100 and 50 ppm CO_2. Additionally, the CO_2 compensation point (CCP, the carboxylation efficiency (CE) and the maximal assimilation at saturating CO_2 were included. (A) Pearson correlation coefficients for average values per taxon represented as heat map using data from C_3, C_3-C_4 and C_4 taxa, (B) Pearson correlation coefficients for average values per taxon represented as heat map using data only from C_3 and C_3-C_4 taxa. Correlations with $p>0.05$ are marked with a cross.

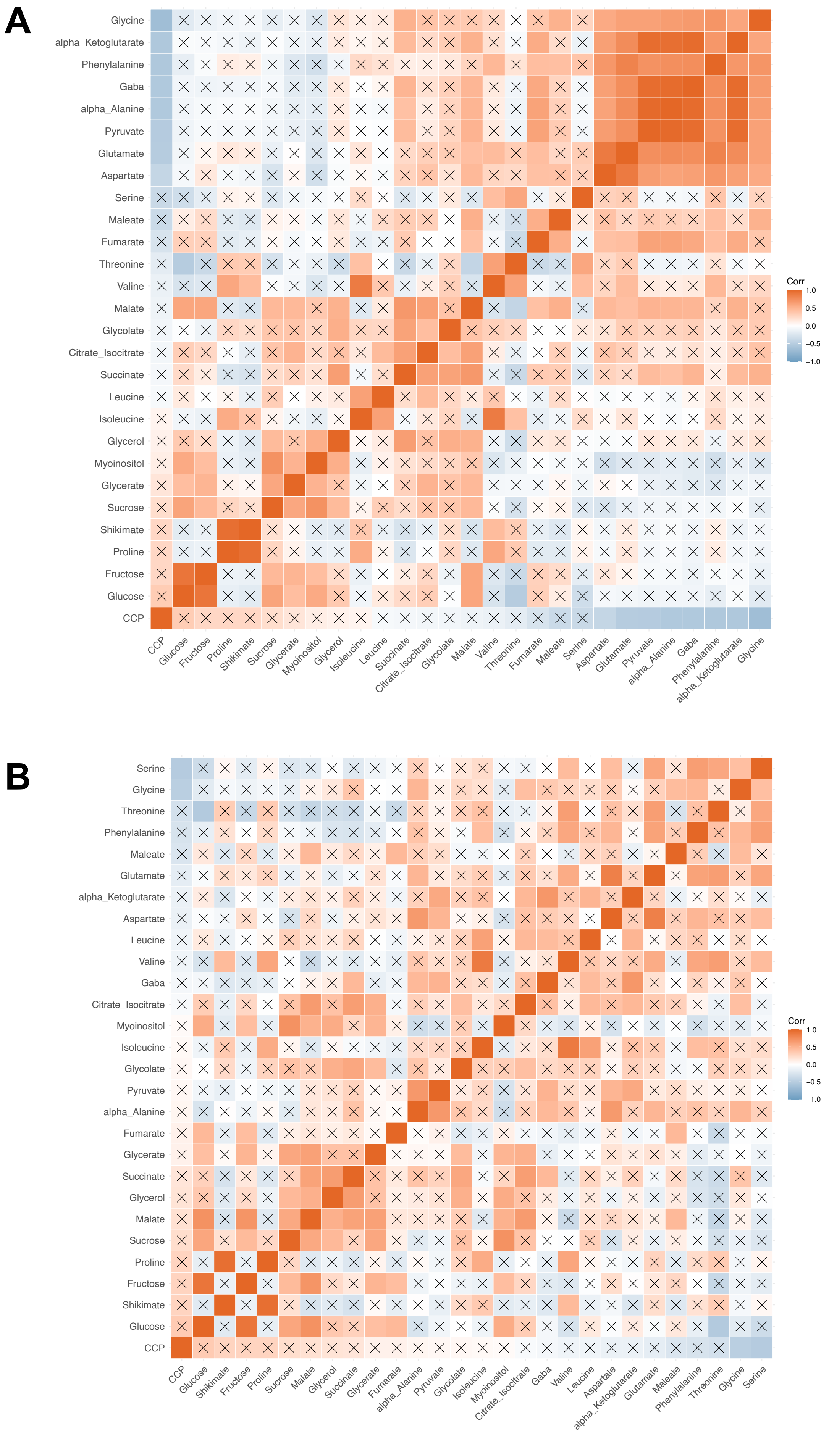
A
Glycine
alpha_Ketoglutarate
Phenylalanine
Gaba
alpha_Alanine
Pyruvate
Glutamate
Aspartate
Serine
Maleate
Fumarate
Threonine
Valine
Malate
Glycolate
Citrate_Isocitrate
Succinate
Leucine
Isoleucine
Glycerol
Myoinositol
Glycerate
Sucrose
Shikimate
Proline
Fructose
Glucose
CCP
CCP Glucose Fructose Proline Shikimate Sucrose Glycerate Myoinositol Glycerol Isoleucine Leucine Succinate Citrate_Isocitrate Glycolate Malate Valine Threonine Fumarate Maleate Serine Aspartate Glutamate Pyruvate alpha_Alanine Gaba Phenylalanine alpha_Ketoglutarate Glycine
Corr
1.0
0.5
0.0
−0.5
−1.0
B
Serine
Glycine
Threonine
Phenylalanine
Maleate
Glutamate
alpha_Ketoglutarate
Aspartate
Leucine
Valine
Gaba
Citrate_Isocitrate
Myoinositol
Isoleucine
Glycolate
Pyruvate
alpha_Alanine
Fumarate
Glycerate
Succinate
Glycerol
Malate
Sucrose
Proline
Fructose
Shikimate
Glucose
CCP
CCP Glucose Shikimate Fructose Proline Sucrose Malate Glycerol Succinate Glycerate Fumarate alpha_Alanine Pyruvate Glycolate Isoleucine Myoinositol Citrate_Isocitrate Gaba Valine Leucine Aspartate alpha_Ketoglutarate Glutamate Maleate Phenylalanine Threonine Glycine Serine
Corr
1.0
0.5
0.0
−0.5
−1.0

Supplemental Figure S3. Correlation matrix of metabolite parameters

Relative concentrations of metabolites were by GC-MS analysis. Additionally the CO_2 compensation point (CCP) was included. (A) Pearson correlation coefficients for average values per taxon represented as heat map using data from C_3, C_3-C_4 and C_4 taxa, (B) Pearson correlation coefficients for average values per taxon represented as heat map using data only from C_3 and C_3-C_4 taxa. Correlations with $p>0.05$ are marked with a cross.

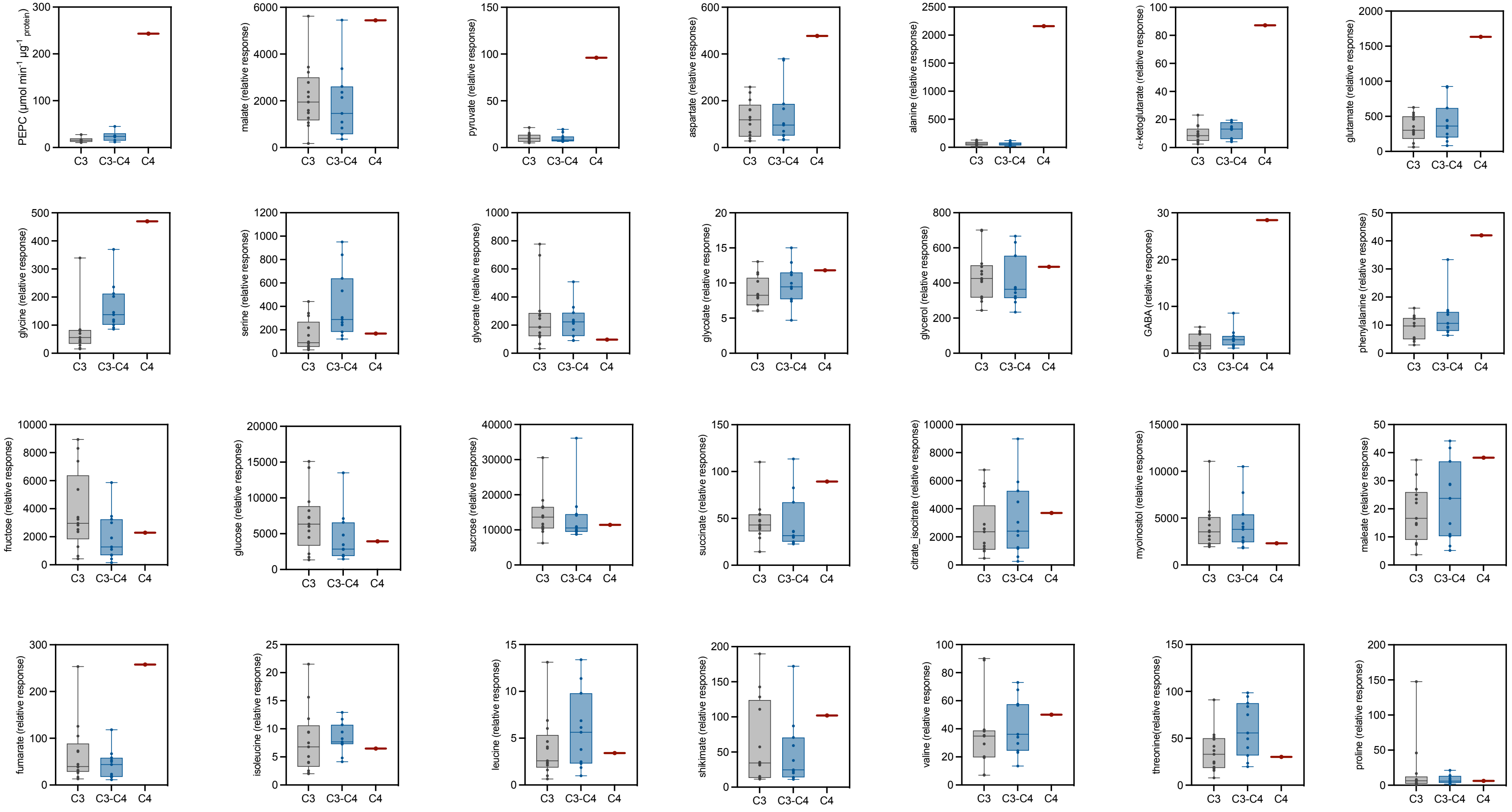

Supplemental Figure S4: Biochemistry data (GC-MS and PEPC activity) sorted by photosynthesis group.
Significant differences between data for the C_3 and C_3-C_4 groups are indicated with stars (*** -> p<0.001; ** -> p<0.01; * -> p<0.05). The C_4 group is represented only by one line from the Cleomaceae (*G. gynandra*).

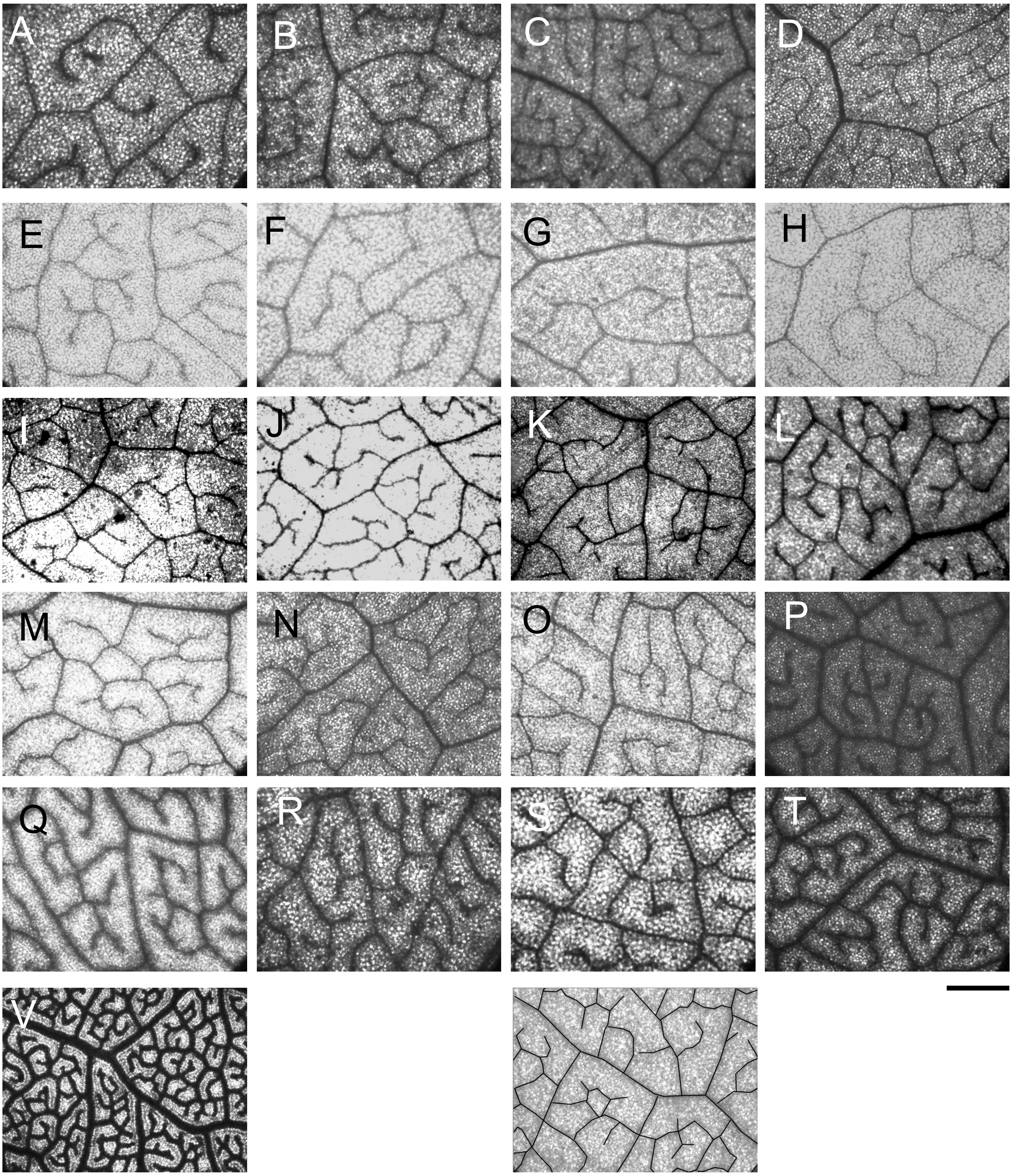
A
B
C
D
E
F
G
H
I
J
K
L
M
N
O
P
Q
R
S
T
V

Supplemental Figure S5. Vein pattern in de-stained leaves

The top free three rows represent C_3 taxa (A, *Brassica repanda; B, Eruca sativa;* C, *Moricandia moricandioides; D, Diplotaxis viminea*; E, *Brassica tournefortii; F, Brassica napus; G Brassica oleraceae; H Brassica rapa*; I, *Hirschfeldia incana* HIR1; J, *Diplotaxis acris; K, Sinapis alba; L, Diplotaxis harra*. The next two rows represent C_3-C_4 classified taxa M, *Brassica gravinae* 3; N, *Diplotaxis erucoides; O, Diplotaxis muralis; P, Hirschfeldia incana* HIR3; Q, *Diplotaxis tenuifolia; R, Moricandia arvensis; S, Moricandia nitens; T Moricandia suffruticosa.* The last row shows the C_4 species *Gynandropsis gynandra* (V). The bar represents 50 µm.

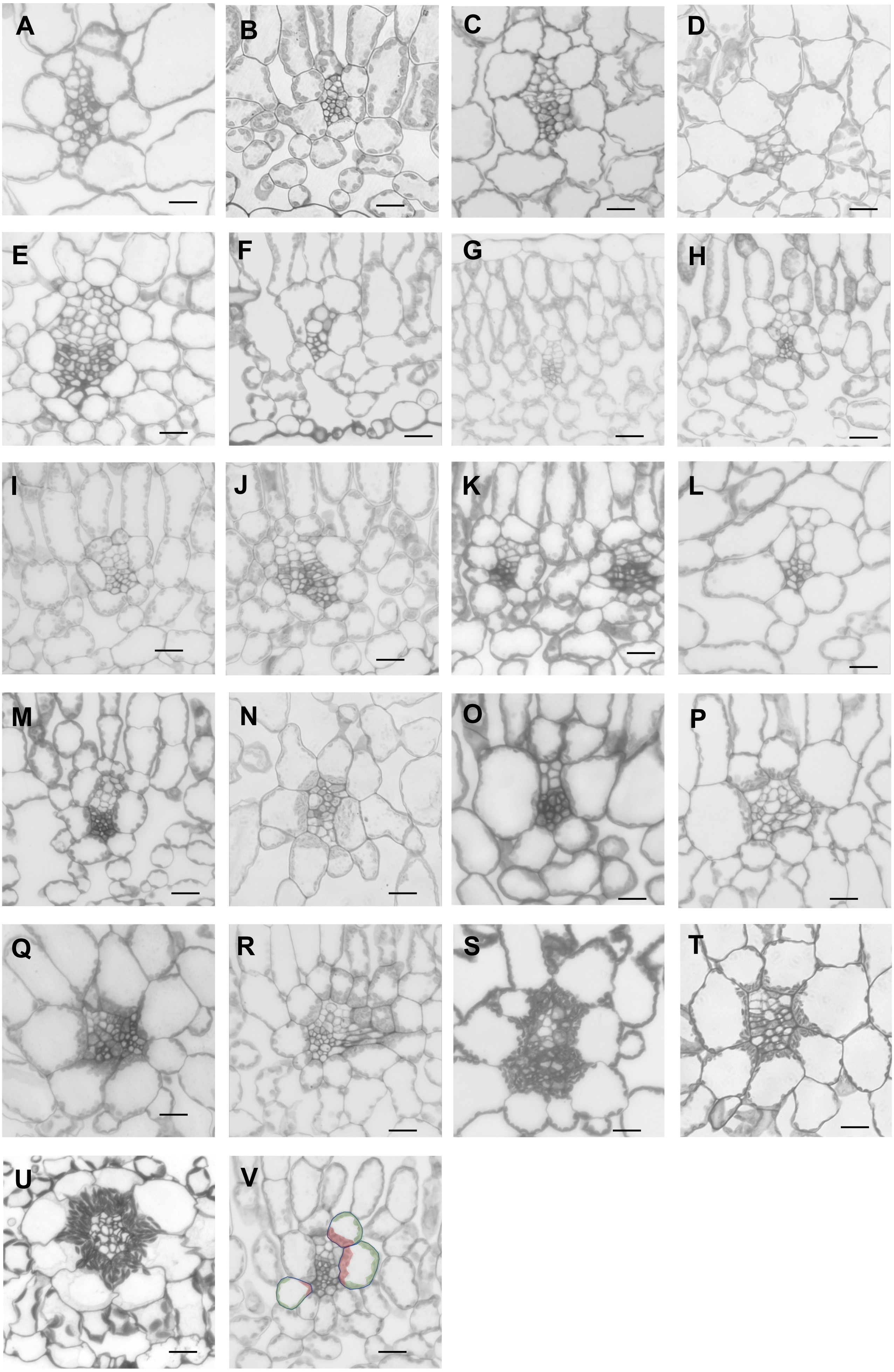
A
B
C
D
E
F
G
H
I
J
K
L
M
N
O
P
Q
R
S
T
U
V

Supplementary Figure 6. Light microscopy of bundle sheath cross section from selected Brassicaceae

The figures show examples of bundle sheath cross section from plant taxa categorized as C3 (A to L), C3-C4 (M to T) or C4 (U). Figure (V) shows the the parameter determined per bundle sheath: bundle sheath cell area (blue order), area of organelles oriented towards the vein (V_organelles, red), area covered by organelles orientated towards intercellular space and mesophyll (M_organelles, green). Always three bundle sheath cells were measured per bundle sheath. The bar represents 20 µm.

(A) *Brassica repanda*, (B) *Eruca sativa*, (C) *Moricandia moricandioides*, (D) *Diplotaxis viminea*, (E) *Brassica oleraceae*, (F) *Hirschfeldia incana* HIR1, (G) *Sinapis alba* , (H) *Diplotaxis tenuisiliqua*, (I) *Brassica tournefortii* 1, (J) *Brassica juncea*, (K) *Brassica napus*, (L) *Raphanus sativus*, (M) *Hirschfeldia incana* HIR3, (N) *Brassica gravinae* 3, (O) *Diplotaxis muralis*, (P) *Diplotaxis erucoides* 1, (Q) *Moricandia suffruticosa*, (R) *Moricandia arvensis*, (S) *Moricandia sinapis*, (T) *Diplotaxis tenuifolia*, (U) *Gynandropsis gynandra*.

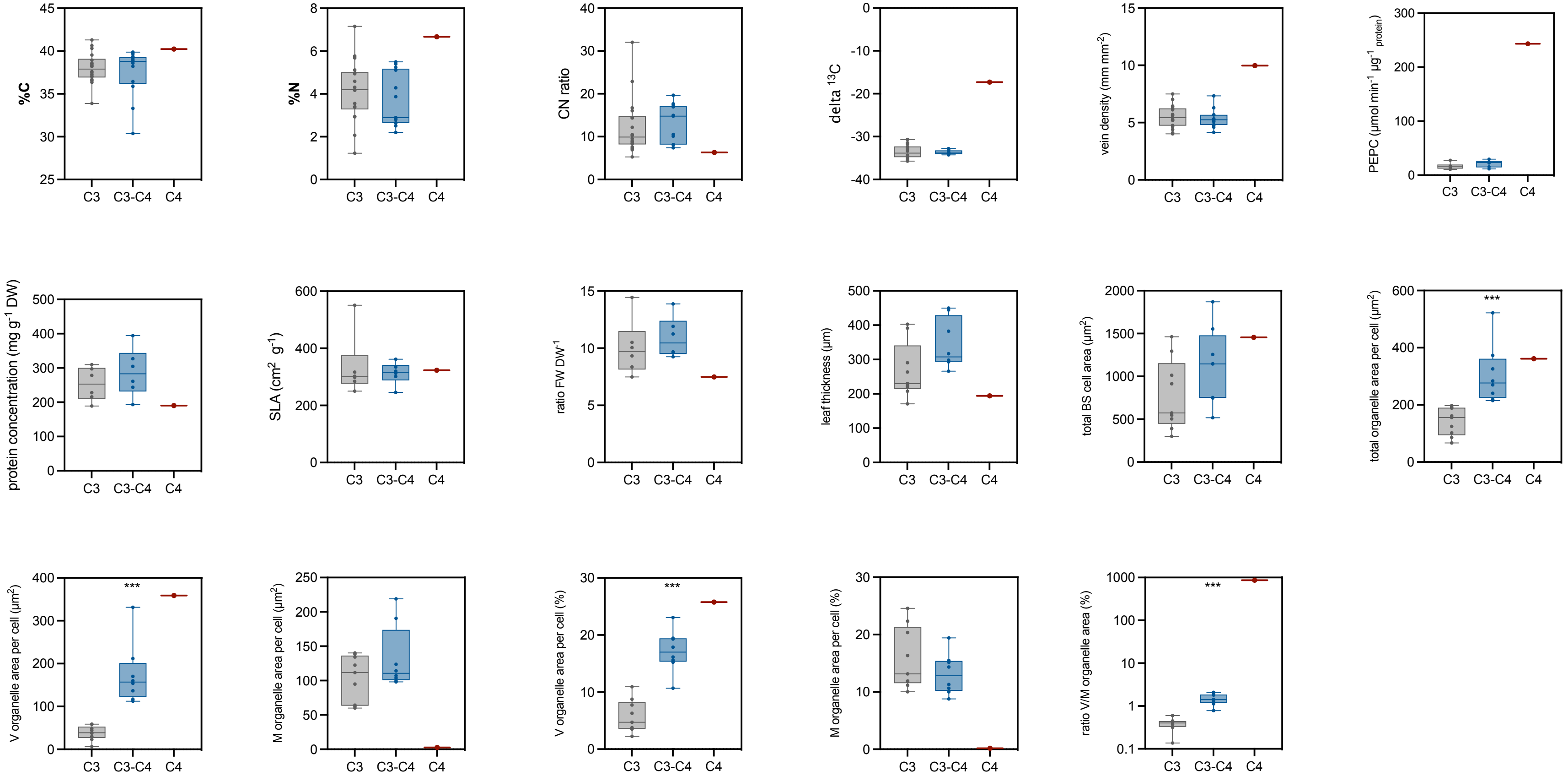

Supplemental Figure S7: Structural data (LM, EA-IRMS) sorted by photosynthesis group.
Significant differences between data for the C_3 and C_3-C_4 groups are indicated with stars (*** -> p<0.001; ** -> p<0.01; * -> p<0.05). The C_4 group is represented only by one line from the Cleomaceae (*G. gynandra*).

A

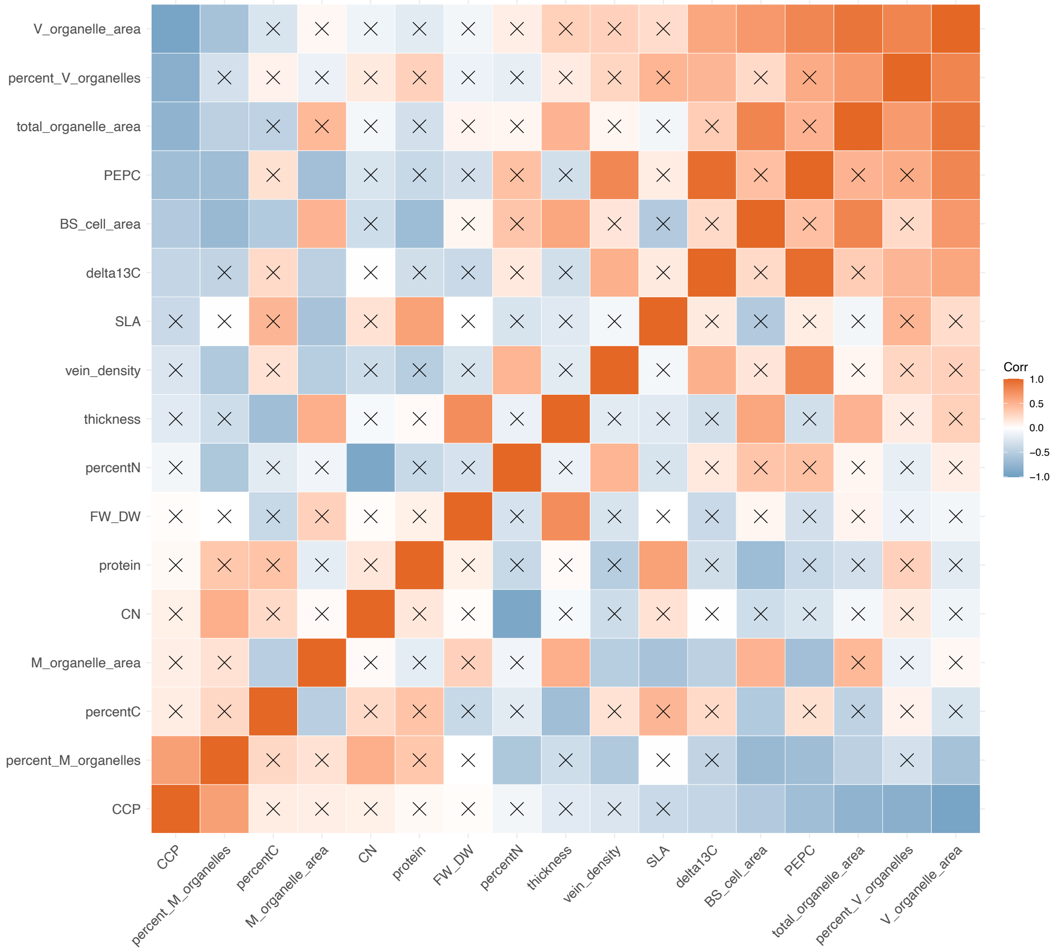
V_organelle_area
percent_V_organelles
total_organelle_area
PEPC
BS_cell_area
delta13C
SLA
vein_density
thickness
percentN
FW_DW
protein
CN
M_organelle_area
percentC
percent_M_organelles
CCP
CCP
percent_M_organelles
percentC
M_organelle_area
CN
protein
FW_DW
percentN
thickness
vein_density
SLA
delta13C
BS_cell_area
PEPC
total_organelle_area
percent_V_organelles
V_organelle_area
Corr
1.0
0.5
0.0
−0.5
−1.0

B

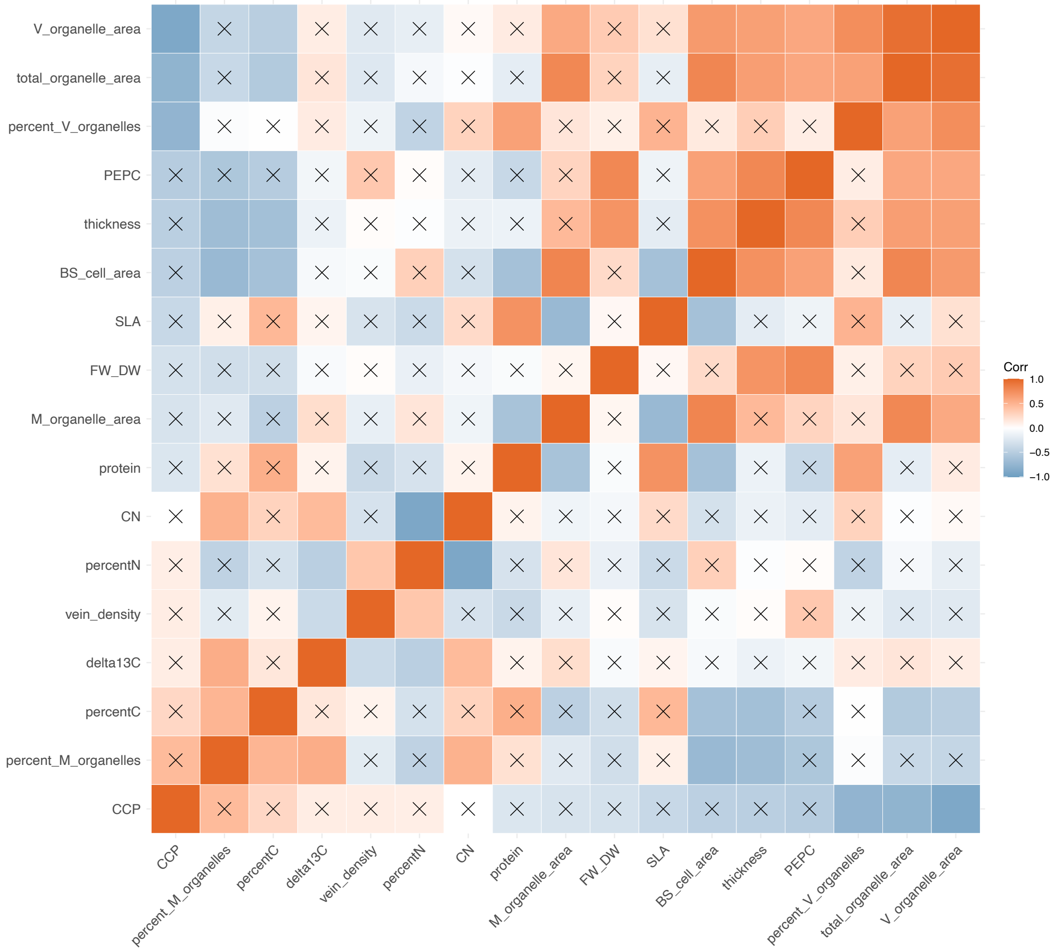
V_organelle_area
total_organelle_area
percent_V_organelles
PEPC
thickness
BS_cell_area
SLA
FW_DW
M_organelle_area
protein
CN
percentN
vein_density
delta13C
percentC
percent_M_organelles
CCP
CCP
percent_M_organelles
percentC
delta13C
vein_density
percentN
CN
protein
M_organelle_area
FW_DW
SLA
BS_cell_area
thickness
PEPC
percent_V_organelles
total_organelle_area
V_organelle_area
Corr
1.0
0.5
0.0
−0.5
−1.0

Supplemental Figure S8. Correlation matrix of leaf structural parameters and PEPC activity

Leaf structural parameters analyzed by EA-IRMS and light microscopy. Additionally, protein concentration and PEPC activity in the leaf were measured, and the CO_2 compensation point (CCP) was included. (A) Pearson correlation coefficients for average values per plant line represented as heat map using data from C_3, C_3-C_4 and C_4 lines, (B) Pearson correlation coefficients for average values per plant line represented as heat map using data only from C_3 and C_3-C_4 lines. Correlations with $p>0.05$ are marked with a cross.
